# A novel detection method for the pathogenic *Aeromonas hydrophila* expressing *aerA* gene and/or *hlyA* gene based on dualplex RAA and CRISPR/Cas12a

**DOI:** 10.3389/fmicb.2022.973996

**Published:** 2022-10-07

**Authors:** Ziqin Lin, Jinfang Lu, Sihong Wu, Xi Lin, Laibao Zheng, Yongliang Lou, Xingxing Xiao

**Affiliations:** Wenzhou Key Laboratory of Sanitary Microbiology, Key Laboratory of Laboratory Medicine, Ministry of Education, School of Laboratory Medicine and Life Sciences, Wenzhou Medical University, Wenzhou, Zhejiang, China

**Keywords:** *Aeromonas hydrophila*, dualplex recombinase-aided amplification assay, CRISPR/Cas12a, detection, diagnosis

## Abstract

*Aeromonas hydrophila* is an emerging waterborne and foodborne pathogen with pathogenicity to humans and warm water fishes, which severely threatens human health, food safety and aquaculture. A novel method for the rapid, accurate, and sensitive detection of pathogenic *A. hydrophila* is still needed to reduce the impact on human health and aquaculture. In this work, we developed a rapid, accurate, sensitive, and visual detection method (dRAA-CRISPR/Cas12a), without elaborate instruments, integrating the dualplex recombinase-assisted amplification (dRAA) assay and CRISPR/Cas12a system to detect pathogenic *A. hydrophila* expressing *aerA* and/or *hlyA* virulence genes. The dRAA-CRISPR/Cas12a method has high sensitivity, which can rapidly detect (about 45 min) *A. hydrophila* with the limit of detection in 2 copies of genomic DNA per reaction, and has high specificity for three pathogenic *A. hydrophila* strains (*aerA^+^hlyA^−^*, *aerA^−^hlyA^+^*, and *aerA^+^hlyA^+^*). Moreover, dRAA-CRISPR/Cas12a method shows satisfactory practicability in the analysis of the spiked human blood and stool and fish samples. These results demonstrate that our developed pathogenic *A. hydrophila* detection method, dRAA-CRISPR/Cas12a, is a promising potential method for the early diagnosis of human *A. hydrophila* infection and on-site detection of *A. hydrophila* in food and aquaculture.

## Introduction

*Aeromonas hydrophila* is a widespread aquatic and zoonotic pathogen (Daskalov, 2006; Li et al., 2011). In animals, *A. hydrophila* mainly causes diseases in aquaculture animals, such as the motile Aeromonas septicemia and red sore disease in warm water fishes (Janda and Abbott, 2010), resulting in huge economic losses and food safety risks. In humans, *A. hydrophila* was initially thought to be an opportunistic pathogen for immunocompromised populations (Balsalobre et al., 2009), but a growing body of research indicates that it is an emerging enteric (Özbaş et al., 2000), waterborne (Chauret et al., 2001), and foodborne (Daskalov, 2006) pathogen regardless of the immune status of the host (Balsalobre et al., 2009).

The pathogenicity of *A. hydrophila* is closely related to its production of virulence factors (Janda and Abbott, 2010; Tichoniuk et al., 2010; Li et al., 2011). It has been reported that the pathogenic *A. hydrophila* can survive and multiply in water, soil and various foods (milk, fish, raw meat, etc.) at room temperature and low temperature (Balakrishna et al., 2010; Janda and Abbott, 2010), and produce virulence factors aerolysin and/or hemolysin (Wong et al., 1998; Heuzenroeder et al., 1999; Balakrishna et al., 2010; Janda and Abbott, 2010). Patients infected with *A. hydrophila* can develop sepsis and necrotizing fasciitis, which have an acute onset and rapid progression (Tsai et al., 2015; Sun et al., 2021) and are life-threatening, mainly through ingestion of contaminated water and food and exposed wounds, respectively (Meng et al., 2015); moreover, sepsis can reach a state of systemic toxicity within 24 h (Zhu et al., 2021). In view of these aspects, a rapid, accurate, and sensitive detection method of pathogenic *A. hydrophila* would contribute to improving the treatment and control strategies and then reducing the hazard of this bacterial infection.

Nowadays, many methods can be used for the detection of *A. hydrophila*. The traditional culture method is accurate, but it takes a long time. The biochemical test is complicated, and only typical strains can be detected because of the limited biochemical characteristics (Abbott et al., 2003). Enzyme-linked immunosorbent assay (ELISA; Swain et al., 2003), dot blotting (Longyant et al., 2010), and serotyping (Nielsen et al., 2001) have low diagnostic sensitivity. Over the past decade, a large number of assays based on PCR technology have been developed to detect *A. hydrophila* by targeting virulence gene (Wang et al., 2003; Hussain et al., 2014). However, these methods require professional personnel and specialized equipment and are not suitable for on-site application or resource-constrained areas. In recent years, isothermal nucleic acid amplification technologies, such as recombinase-aided amplification (RAA; Piepenburg et al., 2006; Subsoontorn et al., 2020) and loop-mediated isothermal amplification (Nagamine et al., 2002), have promoted the development of nucleic acid amplification without thermal cyclers. RAA assay that only requires one primer set and can be reacted even at body temperature of operator (Wang et al., 2017) has been used for the detection of pathogens such as *A. hydrophila* (Qu et al., 2021) and *Enterocytozoon hepatopenaei* (Zhou et al., 2020), but this method has low sensitivity (Xiao et al., 2021) and the result is complicated to obtain (Zhou et al., 2020).

Recently, the discovery of the trans-cleavage activity of clustered regularly interspaced short palindromic repeats (CRISPR)-associated protein Cas12a has made the CRISPR/Cas12a system a hot spot in the field of *in vitro* diagnostics (Chen et al., 2018; Li et al., 2018a,b). Chen et al. developed a DETECTR platform, consisting of a RPA assay and the CRISPR/Cas12a system, with aM sensitivity and high specificity to distinguish between HPV16 and HPV18 (Chen et al., 2018). Zhang et al. used PCR assay and CRISPR/Cas12a system to develop an integrated naked-eye detection method for the *tlh* gene of *Vibrio parahaemolyticus* with a detection limit of 1.02 × 10^2^ copies/μl (Zhang et al., 2020). Because of its speed, sensitivity, specificity, and simplicity, DETECTR platform is a promising method to be used for on-site detection, and has been performed to detect pathogens including *Listeria monocytogenes* (Li et al., 2021), *Vibrio vulnificus* (Xiao et al., 2021), and SARS-CoV-2 (Broughton et al., 2020).

**Figure 1:**
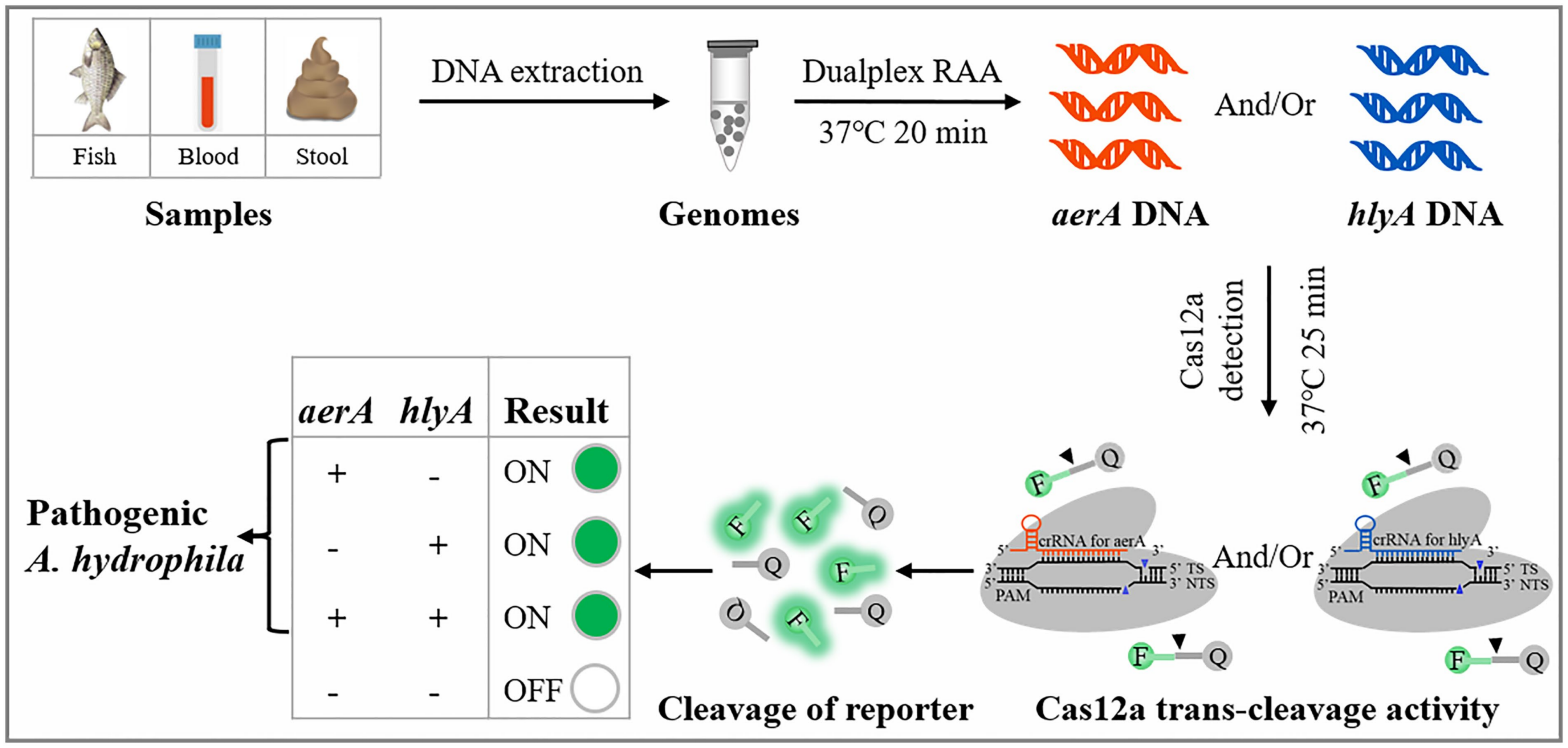
Schematic illustration of the developed dRAA-CRISPR/Cas12a method in the detection of *pathogenic Aeromonas hydrophila*.

The virulence factors play roles through cooperation or alone in the establishment of *A. hydrophila* infection (Nawaz et al., 2010; Li et al., 2011; Nagar et al., 2011; Igbinosa and Okoh, 2013), and the detection methods only targeting one virulence gene of pathogenic *A. hydrophila* always lead to false negative results (Elsheshtawy et al., 2019). Here, a dRAA-CRISPR/Cas12a method targeting *aerA* and *hlyA* genes for the detection of pathogenic *A. hydrophila* without elaborate instruments was developed by integrating dualplex RAA (dRAA) and CRISPR/Cas12a system (Figure 1), and was compared with the dPCR-CRISPR/Cas12a method to investigate their sensitivity in detecting *A. hydrophila* stains and practicability in spiked samples. The whole process only takes 45 min, and the sensitivity is as low as 2 copies per reaction. The dRAA-CRISPR/Cas12a method we developed may be a promising method for rapid, accurate, and sensitive detection of pathogenic *A. hydrophila* in samples from human, fish, and food.

## Materials and methods

### Bacterial strains

Bacterial strains preserved in our laboratory were used in this study and consisted of eight standard strains and eight isolation strains. The standard strains were *A. hydrophila* (ATCC 7966), *Vibrio vulnificus* (ATCC 27562), *Vibrio alginolyticus* (ATCC 17749), *Vibrio harvey* (ATCC 14126), *Staphylococcus aureus* (ATCC 25923), *Escherichia coli* (ATCC 25922), *Pseudomonas aeruginosa* (ATCC 27853) and *Bacillus cereus* (ATCC 14579). The isolation strains were *A. hydrophila* strain 1, *A. hydrophila* strain 2, *A. hydrophila* strain 3, *A. veronii*, *A. sobria*, *Vibrio parahaemolyticus*, *Edwardsiella piscicida*, and *Salmonella typhimurium*, which were isolated from affected aquatic animal, patients, and environment. The *aerA* and *hlyA* genes expressed in these four *A. hydrophila* strains were detected by dualplex RAA assay, and the results showed that *A. hydrophila* strain 1 (NQ201810) is *aerA^−^hlyA^−^*, *A. hydrophila* strain 2 (NQ201906) is *aerA^+^hlyA^−^*, *A. hydrophila* strain 3 (AS1.1801) is *aerA^−^hlyA^+^*, and *A. hydrophila* strain 4 (ATCC 7966) is *aerA^+^hlyA^+^* (Supplementary Figure S1). Moreover, the expression of *aerA* and *hlyA* genes in *A. veronii* and *A. sobria* was also detected by dualplex RAA assay, and the results showed that these two species of *Aeromonas* are *aerA^+^hlyA^−^* (Supplementary Figure S2).

### Genomic DNA extraction

Bacterial strains were inoculated into 2216E broth or LB medium and cultured with shaking at 28°C or 37°C for 12–16 h, and then the genomic DNA of them were extracted by Kit-based and NaOH-based method. The Kit-based method for the extraction of genomic DNA was conducted with a MiniBEST Bacteria Genomic DNA Extraction Kit Ver.3.0 (9,763; TaKaRa Bio Inc., Japan) according to the Kit manual. The NaOH-based method was conducted to extract the genomic DNA of *A. hydrophila* according to the method mentioned in two published papers (Zhang et al., 2020; Xiao et al., 2021). The genomic DNA was stored at −20°C and then used as the templates of RAA and PCR assays.

### Nucleic acid preparation

The primer sets for *aerA* (F: 5′-AAGACGGCCATCAAGGTCAG-3′; R: 5′- ACGAAGGTGTGGTTCCAGT-3′) and *hlyA* (F: 5′-CCGGCATCTCTTTTGATGCG-3′; R: 5′-GGATGTTGACCGAGGAGTCG-3′) were used to amplify the *aerA* gene and *hlyA* gene of *A. hydrophila* by PCR assay respectively, and then the PCR products were sequenced by GENEWIZ (GENEWIZ, China).

Several sequences of *Aeromonas aerA* and *hlyA* genes obtained from GenBank were aligned using the online tool, Clustal Omega. The aligned results and the conserved regions of *A. hydrophila aerA* and *hlyA* genes were shown in Supplementary Figures S4, S5, respectively. The regions of *aerA* and *hlyA* sequences that were highly conserved among *A. hydrophila* were selected as templates for the design of RAA primers, which was conducted with NCBI Primer-BLAST. Five primer sets for *aerA* gene and four primer sets for *hlyA* gene were designed according to the design instruction of RPA primer^1^, and the sequences of them were listed in Supplementary Table S1.

According to the complementary pairing characteristics of crRNA with target sequence (Chen et al., 2018), PAM sequences (TTTN) were first found from RAA amplicon of *aerA* or *hlyA*, and then 15–25 bp was selected from the adjacent sequence of PAM as the target sequence. The specific sequences that can distinguish *A. hydrophila* from other pathogens were selected from the candidate target sequences (Supplementary Figures S6, S7), and the crRNA sequence was comprised of the repeat sequence (UAA UUU CUA CUA AGU GUA GAU) and the selected specific sequence. The ssDNA-FQ reporter (5′−/6-FAM/TTATT/BHQ1/−3′) was used to indicate the existence of the target gene. The crRNA and ssDNA-FQ were purchased from GENEWIZ (GENEWIZ, China).

The genomic DNA of three pathogenic *A. hydrophila* strains (*aerA^+^hlyA^−^*, *aerA^−^hlyA^+^*, and *aerA^+^hlyA^+^*) extracted by Kit-based method were gradiently diluted with 1 × NEB buffer 2.1, and the obtained diluents of *A. hydrophila* genomic DNA with different concentrations (1 × 10^0^ to 1 × 10^7^ copies/μl) were stored at −80°C.

### Single and dualplex RAA assay

The RAA Nucleic Acid Amplification Kit (B00000; Jiangsu Qitian Gene Biological Co., China) was employed to conduct single and dualplex RAA assay. Single RAA assay was carried out according to the Kit manual. Briefly, 25 μl of buffer V, 16.5 μl of purified water, 2 μl of primer F (10 μm), 2 μl of primer R (10 μm), 2.5 μl of magnesium acetate, and 2 μl of genomic DNA were added to the reaction tube. After being softly vortexed for 8 s, the reaction tube was incubated for 20 min at a 37°C water bath. The procedure for dRAA assay was similar to single RAA assay, and the only difference was that the addition volume of each primer (20 μm) in dRAA assay was 1 μl.

### Dualplex RAA-CRISPR/Cas12a assay

The product of dRAA assay was used as the target of CRISPR-Cas12a system. The Cas12a trans-cleavage reaction was performed as follows: 5 μl of 1,000 nm Cas12a (M0653T; New England Biolabs Inc., MA, United States) and 5 μl of 400 nm crRNAmix (crRNA mixture), consisting of crRNA for *aerA* (ACR) and crRNA for *hlyA* (HCR), were preincubated at 37°C for 20 min to form Cas12a-crRNA complex. 10 μl of 1,000 nm ssDNA-FQ, 10 μl of 1 × NEB buffer 2.1, and 2 μl of dRAA product were added to the tube containing 10 μl of Cas12a-crRNA complex. After softly vortexed for 8 s, the tube containing 32 μl of mixture was incubated at 37°C for 35 min. The results can be read with an UV flashlight or a multifunctional microplate reader (*λ*_ex_: 485 nm and *λ*_em_: 520 nm). In this study, we optimized the concentration of Cas12a, the concentration of ssDNA-FQ, and the Cas12a cleavage time.

### Dualplex PCR-CRISPR/Cas12a assay

The procedure for dPCR-CRISPR/Cas12a assay was similar to the dRAA-CRISPR/Cas12a assay. Briefly, dualplex PCR assay was carried out using Phanta® Max Super-Fidelity DNA Polymerase (P505-d1; Vazyme Biotechnology Co. LTD., China) in a 50 μl reaction mixture, containing 25 μl of 2 × Phanta Max Buffer, 1 μl of 10 μm forward and reverse primers (total of 4 μl; Supplementary Table S1), 1 μl of dNTP Mix (10 mm each), 1 U Phanta Max Super-Fidelity DNA Polymerase, 2 μl of genomic DNA, and 17 μl of H_2_O. 2 μl of dPCR product was then added to the reaction mixture containing the ssDNA-FQ and Cas12a-crRNA complex.

### Spiked sample testing

This experiment was conducted by two operators. Firstly, 15 healthy crucian carps were purchased from supermarket. One operator took out the livers of crucian carp, cut them into small pieces, and then added 20 mg of tissue to the 15 tubes containing 200 μl of 0.5 M NaOH solution at clean workbench. Then, 15 tubes were numbered, and 1 × 10^3^ CFU of *A. hydrophila* were added to some tubes. These tissue samples were grinded with a Disposable Tissue Grinding Pestle (Sangon, China) for 3 min, and upon 20-fold dilution with H_2_O, 2 μl of lysate was used as the DNA template for the dRAA assay. Above was the NaOH-based method for the extraction of *A. hydrophila* genomic DNA from spiked fish (Zhang et al., 2020; Xiao et al., 2021; Zhao et al., 2022), and meanwhile, the Kit-based extraction method was also conducted as a comparative test. The other operator who did not know the number of spiked samples performed the dRAA-CRISPR/Cas12a assay.

### Application of dRAA-CRISPR/Cas12a assay for human blood and stool specimens

Human blood and stool samples were provided by three healthy volunteers. The spiked samples were prepared by adding 100 μl of human blood or 200 mg of stool into the tubes containing 1 × 10^3^ CFU of *A. hydrophila* (*aerA^+^hlyA^+^*). The MiniBEST Universal Genomic DNA Extraction Kit (9,765; TaKaRa Bio Inc., Japan) and the TIANamp Stool DNA Kit (DP328; TIANGEN BIOTECH CO. LTD, China) were employed to extract genomic DNA from these spiked samples and samples without *A. hydrophila*, which acted as negative controls. The genomic DNA of these samples were then detected by dRAA-CRISPR/Cas12a assay.

### Statistical analysis

Statistical analysis was performed using SPSS 13.0 software (SPSS Inc., Chicago, IL, USA). The data were analyzed by Student’s *t* test. *p* < 0.05 (indicated by *) was considered statistically significant.

## Results

### Screening the RAA primer pairs targeting *aerA* gene and *hlyA* gene

**Figure 2:**
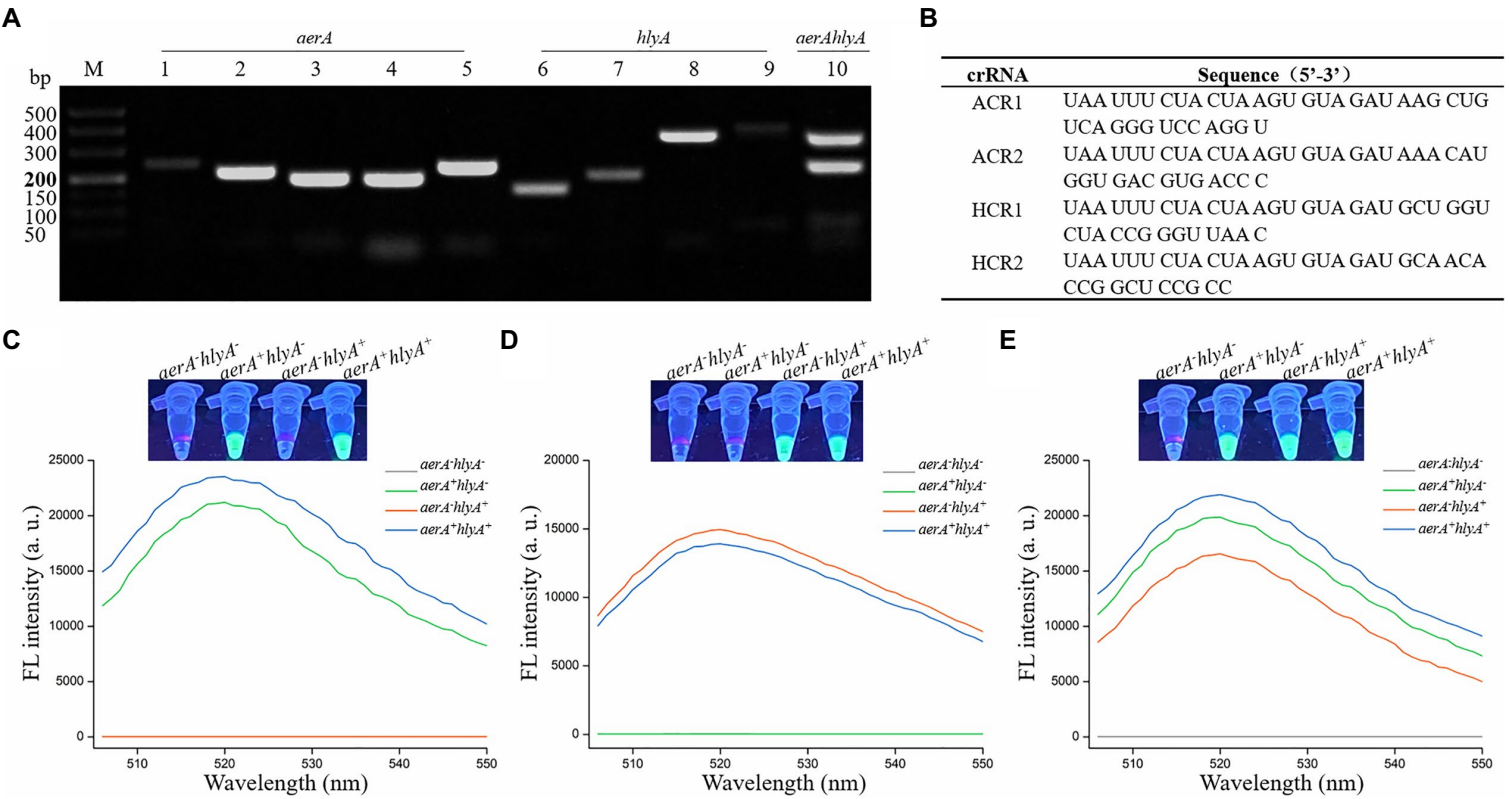
Feasibility verification of the dRAA-CRISPR/Cas12a assay in the detection of *Aeromonas hydrophila*. **(A)** Screening the RAA primer pairs. The single and dualplex RAA assays were performed using the genomic DNA extracted from *aerA^+^hlyA^+^ A. hydrophila* strain as the template. Lanes 1–5, screening the primer pair targeting for *aerA* gene. Lanes 6–9, screening the primer pair targeting for *hlyA* gene. Lane 10, feasibility of dRAA assay with the optimal *aerA* and *hlyA* primer pairs. **(B)** Sequences of crRNA used in this study. **(C–E)** Feasibility verification of the dRAA-CRISPR/Cas12a assay using the ACR1 and ACR2 **(C)**, HCR1 and HCR2 **(D)**, and ACR1, ACR2, HCR1 and HCR2 **(E)** in the detection of four *A. hydrophila* strains. Fluorescence signals were read using an UV flashlight (upper) or a multifunctional microplate reader (below).

High amplification efficiency of the amplification assay can increase the detection sensitivity of the method (Clementi et al., 1993; Pfaffl, 2004). To obtain the primer pair with high amplification efficiency, we designed five primer pairs and four primer pairs targeting *aerA* gene and *hlyA* gene, respectively, and then screened an optimal primer pair according to the relative intensity of RAA product band for each primer pair. The results showed that all the predicted product bands were clearly distinguishable (Figure 2A). As for the *aerA* gene, the band intensity of the No.5 lane obtained with the primer set AF5 (5′-GCCATCAAGGTCAGCAATTTTGCGTACAAC-3′)/AR5 (5′- CACTTGAACTTGTTCTTGGTGGTCACCTTCTC-3′) was the strongest among the five bands, while as for the *hlyA* gene, it was the No.8 lane obtained with the primer set HF3 (5′- CACGTGGCCTTCTACCTCAACGTCAACC-3′)/HR3 (5′- CCTTGGTGTTGGACGCCTCGATGCTGAA-3′). Furthermore, these two primer sets were used to amplify *aerA* gene and *hlyA* gene through dRAA assay, and the result showed that two predicted bands with satisfactory intensity were present at the No.10 lane. Therefore, the RAA primer sets, AF5/AR5 and HF3/HR3, were chosen as the optimal primers for the dRAA reaction, and the amplicon of AF5/AR5 and HF3/HR3 was used to design crRNA for *aerA* and *hlyA*, respectively.

### Feasibility verification of the dRAA-CRISPR/Cas12a assay in the detection of *Aeromonas hydrophila*

According to the principle of crRNA design mentioned in Materials and Methods, ACR1 and ACR2, targeting *aerA* gene, and HCR1 and HCR2, targeting *hlyA* gene, were designed, and the sequences of these four crRNAs were listed in Figure 2B. To investigate the validity of the designed crRNA and verify the feasibility of the dRAA-CRISPR/Cas12a method, the dRAA-CRISPR/Cas12a assays for the detection of four *A. hydrophila* strains (*aerA^−^hlyA^−^*, *aerA^+^hlyA^−^*, *aerA^−^hlyA^+^* and *aerA^+^hlyA^+^*) were performed using the ACR1 and ACR2 (Figure 2C), HCR1 and HCR2 (Figure 2D), and ACR1, ACR2, HCR1 and HCR2 (Figure 2E), respectively. As shown in Figure 2C, only the genomic DNA samples extracted from *aerA^+^hlyA^−^* and *aerA^+^hlyA^+^ A. hydrophila* strains could generate fluorescence signals, indicating that ACR1 and ACR2 were valid and specific crRNAs in the detection of *A. hydrophila* expressing *aerA* gene. Our results also indicated that HCR1 and HCR2 were valid and specific crRNAs in the detection of *A. hydrophila* expressing *hlyA* gene (Figure 2D). Furthermore, as shown in Figure 2E, only the genomic DNA samples extracted from *A. hydrophila* expressing *aerA* and/or *hlyA* genes could generate fluorescence signals, indicating that our developed method, dRAA-CRISPR/Cas12a, using ACR1, ACR2, HCR1 and HCR2 as the crRNAmix could be used to detect the pathogenic *A. hydrophila* expressing *aerA* and/or *hlyA* genes.

### Optimization of the conditions of dRAA-CRISPR/Cas12a assay

**Figure 3:**
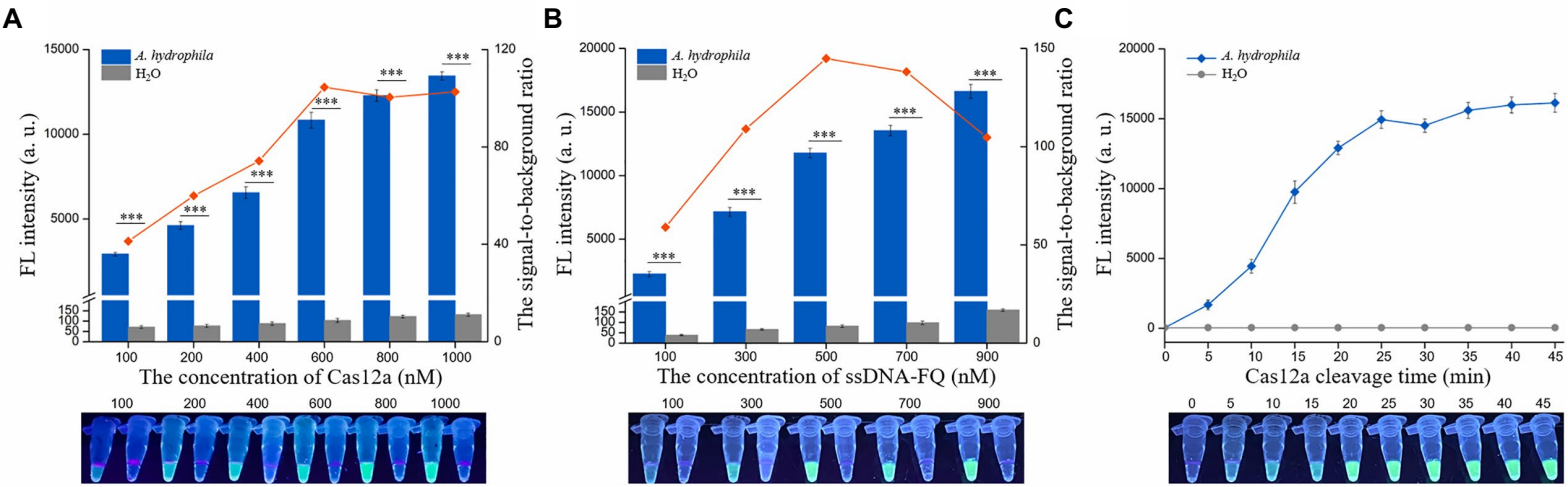
Optimization of the conditions of dRAA-CRISPR/Cas12a assay. The dRAA-CRISPR/Cas12a assays were conducted using AF5/AR5 and HF3/HR3 as RAA primer sets, *aerA^+^hlyA^+^ A. hydrophila* genomic DNA (1 × 10^4^ copies/μl) or H_2_O as a RAA template, and ACR1, ACR2, HCR1 and HCR2 as crRNAmix to optimize the concentration of Cas12a **(A)** and ssDNA-FQ **(B)** and the cleavage time of Cas12a **(C)**. The results were read using an UV flashlight (below) or a multifunctional microplate reader (upper). **(A,B)** *n* = 3 technical replicates; two-tailed Student’s *t* test; ***p < 0.001; bars represent mean ± SEM.

The concentrations of Cas12a and ssDNA-FQ and the Cas12a cleavage time are related to cleavage efficiency, signal output, and reaction speed of the CRISPR-based detection method (Broughton et al., 2020; Sun et al., 2020; Fu et al., 2022). To achieve an ideal reaction performance, dRAA-CRISPR/Cas12a assay was conducted to optimize the concentration of Cas12a, the concentration of ssDNA-FQ and the Cas12a cleavage time with the *aerA^+^hlyA^+^ A. hydrophila* genomic DNA (1 × 10^4^ copies/μL) and H_2_O as templates. The ratio of the fluorescence intensity triggered by *A. hydrophila* (F) to the fluorescence intensity triggered by H_2_O (F_0_) was employed to assess the reaction concentrations of Cas12a and ssDNA-FQ. As shown in Figure 3A, both the F and F_0_ increased with the increasing of the Cas12a concentrations, and when the Cas12a concentration was 600 nm, the ratio of F/F_0_ reached maximum. As shown in Figure 3B, the results indicated that the optimal concentration of ssDNA-FQ was 500 nm. The cleavage time of Cas12a was further explored using the optimized concentrations of Cas12a (600 nm) and ssDNA-FQ (500 nm). The results showed that the increasing of F reached a plateau after 25 min, which indicated that the optimal cleavage time of Cas12a was 25 min. Therefore, the reaction conditions of our developed method, dRAA-CRISPR/Cas12a, were as followed: 20 min of RAA reaction, 5 μl of 600 nm Cas12a, 5 μl of 400 nm crRNAmix, 10 μl of 500 nm ssDNA-FQ, and 25 min of Cas12a cleavage, and in the subsequent experiments, dRAA-CRISPR/Cas12a assays were conducted according to the above conditions.

### Sensitivity of dRAA-CRISPR/Cas12a assay for detecting *Aeromonas hydrophila*

**Figure 4:**
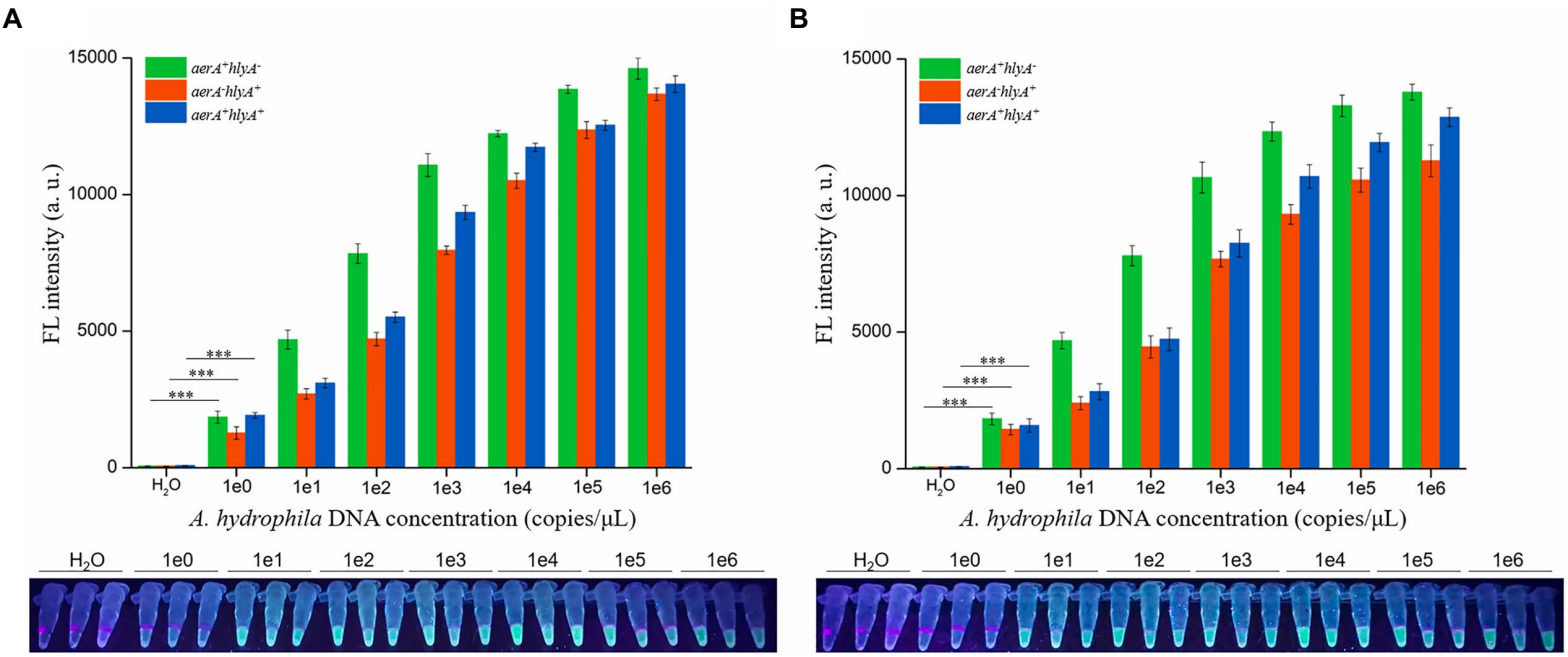
Sensitivity evaluation. Sensitivity of dRAA-CRISPR/Cas12a **(A)** and dPCR-CRISPR/Cas12a **(B)** assays were evaluated in the detection of pathogenic *Aeromonas hydrophila*. The different concentrations (1 × 10^0^–1 × 10^6^ copies/μl) of *A. hydrophila* genomic DNA were used as the detection template, and H_2_O was used as a negative control. The results were read using an UV flashlight (below) or a multifunctional microplate reader (upper). *n* = 3 technical replicates; two-tailed Student’s *t* test; ***p < 0.001, experimental group versus corresponding negative control group (only shown the 1e0 vs. H_2_O); bars represent mean ± SEM.

To investigate the detection sensitivity of dRAA-CRISPR/Cas12a assay for pathogenic *A. hydrophila*, 2 μl of the genomic DNA with different concentrations (1 × 10^0^–1 × 10^6^ copies /μL) extracted from three *A. hydrophila* strains (*aerA^+^hlyA^−^*, *aerA^−^hlyA^+^* and *aerA^+^hlyA^+^*) were used as templates to perform the dRAA assay, and then 2 μl of dRAA product was subjected to Cas12a cleavage test. The dPCR-CRISPR/Cas12a assay was conducted as a comparative test. The results of dRAA-CRISPR/Cas12a assays showed that fluorescence signals were detected in all samples except negative control (H_2_O; Figure 4A), and the limit of detection (LOD) of dRAA-CRISPR/Cas12a assay for each *A. hydrophila* strain reached 2 copies per reaction, which was in line with the LOD of dPCR-CRISPR/Cas12a assay (Figure 4B). Therefore, our developed detection method, dRAA-CRISPR/Cas12a, showed high sensitivity (2 copies/reaction) for pathogenic *A. hydrophila* expressing *aerA* and/or *hlyA* genes.

### Specificity of dRAA-CRISPR/Cas12a assay for detecting *Aeromonas hydrophila*

**Figure 5:**
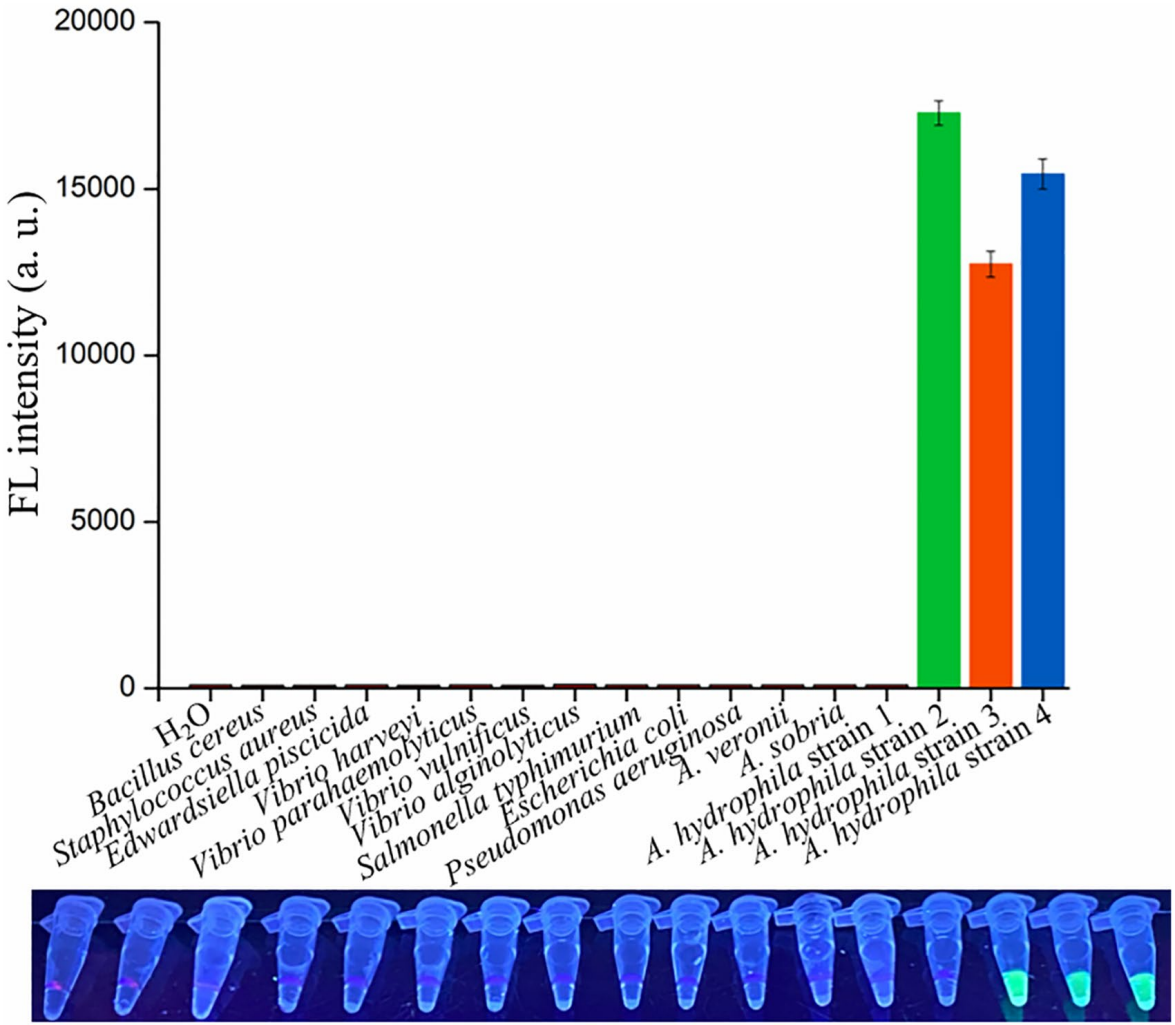
Specificity and practicability evaluation of dRAA-CRISPR/Cas12a assay. Specificity evaluation. 16 bacterial strains were employed to evaluate the specificity of dRAA-CRISPR/Cas12a assay in the detection of *A. hydrophila*. *A. hydrophila* strain 1, 2, 3, and 4 were *aerA^−^hlyA^−^*, *aerA^+^hlyA^−^*, *aerA^−^hlyA^+^*, and *aerA^+^hlyA^+^*, respectively. H_2_O was used as a negative control. *n* = 3 technical replicates; bars represent mean ± SEM.

To evaluate the specificity of dRAA-CRISPR/Cas12a assay in *A. hydrophila* detection, 16 genomic DNA samples extracted from four *A. hydrophila* strains and 12 other strains of waterborne and/or foodborne pathogenic bacteria were used as dRAA templates, and H_2_O was used as negative control. As shown in Figure 5, one *A. hydrophila* strain (*aerA^−^hlyA^−^*) and 12 other bacterial strains did not generate the fluorescence signal, while it was only detected in three *A. hydrophila* strains expressing *aerA* and/or *hlyA* genes, indicating no cross-reactions with non-pathogenic *A. hydrophila* and non-*Aeromonas hydrophila* bacteria of dRAA-CRISPR/Cas12a assay in pathogenic *A. hydrophila* detection. Therefore, our developed detection method, dRAA-CRISPR/Cas12a, showed high specificity for pathogenic *A. hydrophila* expressing *aerA* and/or *hlyA* genes.

### Practicability evaluation of the dRAA-CRISPR/Cas12a assay in the detection of spiked samples

**Figure 6:**
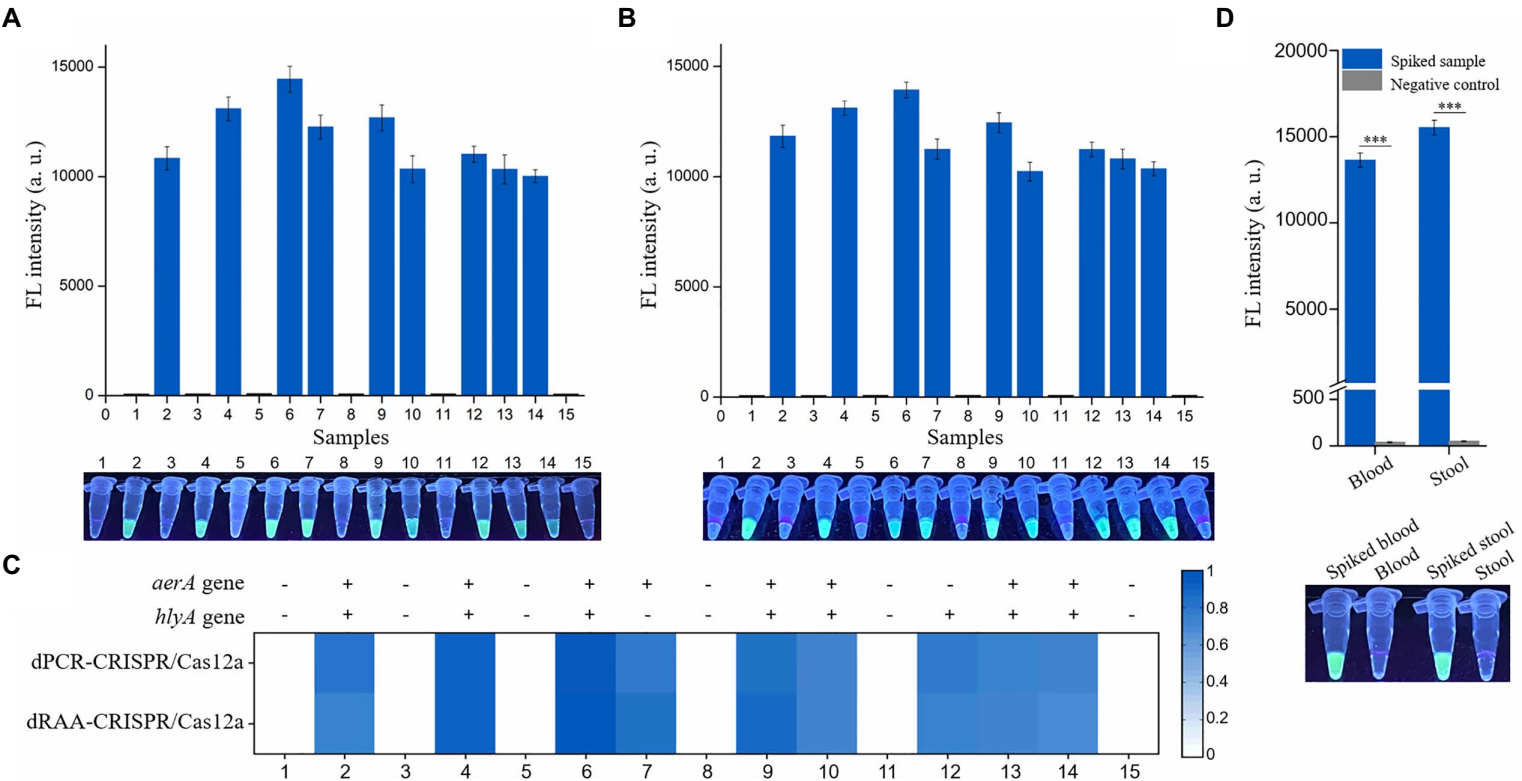
Practicability evaluation of the dRAA-CRISPR/Cas12a and dPCR-CRISPR/Cas12a assays in the detection of spiked samples. Six normal liver samples and nine liver samples spiked with 1 × 10^3^ CFU of *A. hydrophila* were used to extract genomic DNA according to the NaOH-based method. These genomic DNA samples were detected using dRAA-CRISPR/Cas12a **(A)** and dPCR-CRISPR/Cas12a **(B)** assays. *n* = 3 technical replicates; bars represent mean ± SEM. **(C)** Heatmap of the results of the two assays for the detection of *A. hydrophila* from 15 crucian carps. **(D)** Human blood and stool samples were spiked with 1 × 10^3^ CFU of *A. hydrophila* (*aerA^+^hlyA^+^*). Normal blood and stool samples were served as negative controls. The fluorescence signals were read using an UV flashlight (below) or a multifunctional microplate reader (upper). *n* = 3 technical replicates; two-tailed Student’s *t* test; ***p < 0.001; bars represent mean ± SEM.

Finally, to evaluate the practicability of the dRAA-CRISPR/Cas12a assay, fish and human samples were employed to conduct this experiment by two operators. For fish samples, livers were taken out from 15 healthy crucian carps, nine liver samples (20 mg/sample) were spiked with 1 × 10^3^ CFU of *A. hydrophila*, and then the genomic DNA was extracted using the NaOH-based method from these 15 liver samples by one operator, while the other one did the dRAA-CRISPR/Cas12a and dPCR-CRISPR/Cas12a assays with these 15 liver samples. The results of dRAA-CRISPR/Cas12a assays showed that only the spiked samples could generate the fluorescence signal (Figure 6A). This result was consistent with the result obtained from dPCR-CRISPR/Cas12a assays (Figures 6B,C). In addition, the genomic DNA extracted from the other 15 liver samples using Kit-based method were detected by dRAA-CRISPR/Cas12a assay, and the same result with Figure 6A was obtained (Supplementary Figure S3). Therefore, these results indicated that our developed method, dRAA-CRISPR/Cas12a, could rapidly and accurately detect *A. hydrophila* from fish samples.

For human samples, blood and stool samples were collected from three healthy volunteers, and some blood and stool were spiked with 1 × 10^3^ CFU of *A. hydrophila* (*aerA^+^hlyA^+^*). As shown in Figure 6D, only the spiked samples could generate the fluorescence signal. These results indicated that our developed method, dRAA-CRISPR/Cas12a, could be used to detect clinical sepsis and gastroenteritis caused by *A. hydrophila*.

## Discussion

*Aeromonas hydrophila* is an emerging pathogen with pathogenicity to humans and fishes (Tichoniuk et al., 2010; Li et al., 2011). Increasingly, *A. hydrophila* is posing a serious threat to human health, aquaculture and food safety, and foodborne outbreaks of its infection have occurred in many countries (Morena et al., 1993; Krovacek et al., 1995; Zhang et al., 2012; Tsheten et al., 2016). Many studies have reported that the pathogenicity of *A. hydrophila* is determined by multiple virulence factors (Janda and Abbott, 2010; Tichoniuk et al., 2010; Li et al., 2011), and based on this reason, there are many methods targeting one or more virulence genes to detect pathogenic *A. hydrophila* (Tichoniuk et al., 2010; Uma et al., 2010; Li et al., 2011; Elsheshtawy et al., 2019; Qu et al., 2021). However, these methods have some flaws, such as long operation time, low sensitivity, complicated operation, and missed detection. In this study, to avoid these flaws, we developed a novel method, dRAA-CRISPR/Cas12a, for the detection of pathogenic *A. hydrophila* targeting *aerA* and *hlyA* genes based on dRAA assay and CRISPR/Cas12a system (Figure 1). This method performed according to our optimized conditions only takes 45 min to obtain the accurate result without an elaborate instrument (Figure 3), and the LOD of it is as low as 2 copies of *A. hydrophila* genomic DNA per reaction (Figure 4A).

The target genes, *aerA* and *hlyA*, encoding aerolysin and hemolysin respectively, of our proposed method are two important virulent genes of *A. hydrophila* (Wong et al., 1998; Biscardi et al., 2002; Ørmen et al., 2003; Janda and Abbott, 2010). Aerolysin and hemolysin are most common in clinical and environmental strains of *A. hydrophila* (Wong et al., 1998; González-Rodríguez et al., 2002; Janda and Abbott, 2010) and play key roles through cooperation or alone in the pathogenicity of it (Biscardi et al., 2002; Singh et al., 2008; Nawaz et al., 2010; Igbinosa and Okoh, 2013). Moreover, the study reported by Heuzenroeder et al. indicated that a method targeting *aerA* and *hlyA* genes was a trustworthy approach to detect pathogenic *A. hydrophila* (Heuzenroeder et al., 1999). Therefore, *aerA* and *hlyA* genes were selected for the detection of pathogenic *A. hydrophila* in this study. Because *aerA* and *hlyA* genes are also present in some other species of *Aeromonas*, the published *aerA* and *hlyA* sequences were downloaded from GenBank and aligned to seek the sequences which are highly conserved in *A. hydrophila* but not in other *Aeromonas* species (Supplementary Figures S4–S7), and based on these sequences, we successfully designed two crRNAs for *aerA* and two crRNAs for *hlyA* (Figures 2B–E). Furthermore, the results of specificity experiment indicated that only three *A. hydrophila* strains expressing *aerA* gene and/or *hlyA* gene could be detected by our developed method, dRAA-CRISPR/Cas12a (Figure 5), which further confirmed the validity of our designed crRNA and RAA primer sets.

In addition, we investigated the sensitivity of dRAA-CRISPR/Cas12a in the detection of three pathogenic *A. hydrophila* strains (*aerA^+^hlyA^−^*, *aerA^−^hlyA^+^* and *aerA^+^hlyA^+^*), and found that the LOD for each strain reached 2 copies of genomic DNA per reaction (Figure 4A), which was consistent with the results of dPCR-CRISPR/Cas12a (Figure 4B). However, because the samples used to investigate the sensitivity of dRAA-CRISPR/Cas12a were the pure bacteria, the result of this experiment may not reflect the real sensitivity of dRAA-CRISPR/Cas12a in detecting the clinical and environmental samples with a large number of impurities that may influence the amplification efficiency of RAA assay. Finally, we evaluated the practicability of the dRAA-CRISPR/Cas12a assay in the detection of spiked fish samples. Our proposed method dRAA-CRISPR/Cas12a could only detect the presence of *A. hydrophila* from all the spiked liver samples (Figure 6A), and this result completely matched the results got from dPCR-CRISPR/Cas12a assay (Figures 6B,C), indicating that dRAA-CRISPR/Cas12a method is a promising candidate for on-site *A. hydrophila* detection in fish samples. Because *A. hydrophila* infection mainly causes sepsis and gastroenteritis in humans (González-Serrano et al., 2002; Zhu et al., 2021), we also performed dRAA-CRISPR/Cas12a method to detect the spiked blood and stool samples, and found that the fluorescence signal could only be detected in spiked blood and stool samples (Figure 6D), indicating that the dRAA-CRISPR/Cas12a method can also be used to diagnose patients infected by *A. hydrophila*.

In conclusion, the dRAA-CRISPR/Cas12a method we developed can rapidly, accurately, and sensitively detect pathogenic *A. hydrophila* expressing *aerA* gene and/or *hlyA* gene, and the results can be read using an UV flashlight. It is very beneficial for early diagnosis and on-site detection of *A. hydrophila* infection especially in resource-poor areas. Apart from dRAA-CRISPR/Cas12a method, dPCR-CRISPR/Cas12a used in this study can also be a candidate to detect *A. hydrophila* in Lab.

## Data availability statement

The original contributions presented in the study are included in the article/Supplementary material, further inquiries can be directed to the corresponding authors.

## Author contributions

XX and YL conceived and designed the study. XX and ZL wrote the manuscript. ZL, JL, and SW performed the experiments and analyzed the data. XL and LZ reviewed the manuscript. All authors read and approved the submitted version.

## Funding

This study was supported by the National Natural Science Foundation of China (grant no. 82002117), the Science and Technology Bureau of Wenzhou (grant no. Y20210109), and the Key Discipline of Zhejiang Province in Medical Technology (First Class, Category A).

## Conflict of interest

The authors declare that the research was conducted in the absence of any commercial or financial relationships that could be construed as a potential conflict of interest.

## Publisher’s note

All claims expressed in this article are solely those of the authors and do not necessarily represent those of their affiliated organizations, or those of the publisher, the editors and the reviewers. Any product that may be evaluated in this article, or claim that may be made by its manufacturer, is not guaranteed or endorsed by the publisher.

## Footnotes

^1^ https://www.twistdx.co.uk/support/rpa-assay-design/
