# Supplementary material for: A novel detection method for the pathogenic Aeromonas hydrophila expressing aerA gene and/or hlyA gene based on dualplex RAA and CRISPR/Cas12a
Source: Front Microbiol. 2022 Oct 7;13:973996. doi: 10.3389/fmicb.2022.973996 (PMC9585296; doi:10.3389/fmicb.2022.973996)
Supplement: Supplementary file 1 [file Data_Sheet_1.PDF]

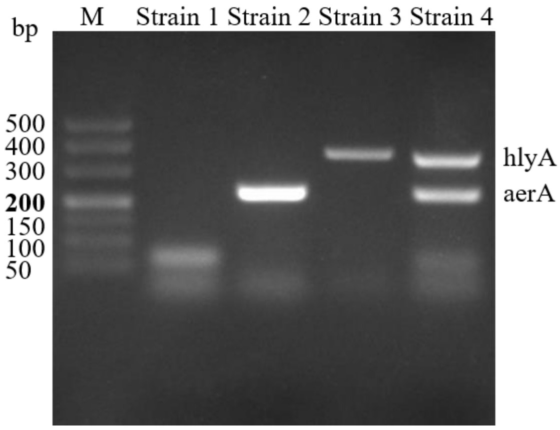

Figure S1. Analysis of the expression of *aerA* and *hlyA* in four *A. hydrophila* strains using dualplex RAA assays. Dualplex RAA assays were conducted using the two primer sets, AF5/AR5 and HF3/HR3.

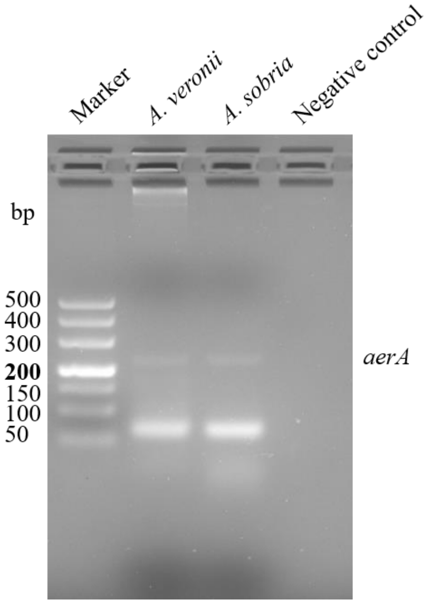

Figure S2. Analysis of the expression of *aerA* and *hlyA* in *A. veronii* and *A. sobria* using dualplex RAA assays. Dualplex RAA assays were conducted using the two primer sets, AF5/AR5 and HF3/HR3.

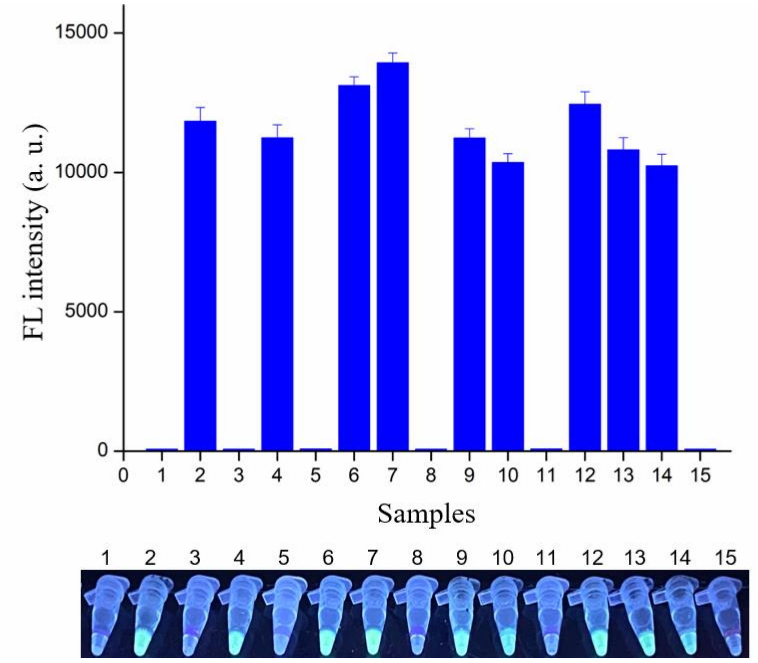

Figure S3. Practicability evaluation of the dRAA-CRISPR/Cas12a assay in the detection of spiked fish samples. Six normal liver samples and nine liver samples spiked with 1 × 10^3 CFU of *A. hydrophila* were used to extract genomic DNA according to the Kit-based method. These genomic DNA samples were detected using dRAA-CRISPR/Cas12a assays. The fluorescence signals were read using an UV flashlight (below) or a multifunctional microplate reader (upper).

Table S1. RAA primer pairs designed in this study.

Primers	Sequences（5’—3’）	Product size（bp）
AF1	ACCAACTGGTCCAAGACCAATACCTATGGC	247
AR1	CATAGGGATAGGAGATGTCAGCCTTGTAGAG	
AF2	CCTGAGCGAGAAGGTGACCACCAAGAACAA	218
AR2	CATAGGGATAGGAGATGTCAGCCTTGTAGAGC	
AF3	CCTGAGCGAGAAGGTGACCACCAAGAACAA	197
AR3	CCTTGTAGAGCTCGATCTTCACCGGGATCTTG	
AF4	GCCATCAAGGTCAGCAATTTTGCGTACAAC	196
AR4	CATAGGTATTGGTCTTGGACCAGTTGGTGG	
AF5	GCCATCAAGGTCAGCAATTTTGCGTACAAC	236
AR5	CACTTGAACTTGTTCTTGGTGGTCACCTTCTC	
HF1	ACCTTCCCGCGCTCGCGCACCTGGAGCCGGGG	158
HR1	TCCAGCGAGATCCGCACTATCTTGGCATCCGG	
HF2	CCTTCTACCTCAACGTCAACCGCAAGATCA	202
HR2	TCCAGCGAGATCCGCACTATCTTGGCATCC	
HF3	CACGTGGCCTTCTACCTCAACGTCAACC	367
HR3	CCTTGGTGTTGGACGCCTCGATGCTGAA	
HF4	CCTTCTACCTCAACGTCAACCGCAAGATCA	397
HR4	CTGTTGAGGTTGGTCGGCAGGCTCTTGA	

Table S2. PCR primer pairs designed in this study.

Primers	Sequences（5’—3’）	Product size（bp）
PCR-AF	AAGACGGCCATCAAGGTCAGCAAT	223
PCR-AR	TGGTCACCTTCTCGCTCAGGCCAT	
PCR-HF	ACCGCAAGATCAGCGATGCCGAGT	334
PCR-HR	TTGGACGCCTCGATGCTGAAGCGGT	

To design RAA primers and crRNA of *aerA* gene, several sequences of *A. hydrophila aerA* gene obtained from GenBank were aligned using the online tool, Clustal Omega (https://www.ebi.ac.uk/Tools/msa/clustalo/). The results were shown in below (**Figure S4**). After analyzed the aligned results, we found that no conserved region can be used to design one crRNA that can target all the *aerA* sequences. Therefore, we designed two crRNAs (ACR1 and ACR2).

```
MF315078.1      ------------------------------------------------------------      0
HQ425626.1      ATGCAAAAACTAAAAATAACTGGCTTGTCATTGATCATATCCGGCCTGCTGATGGCACAG      60
HQ425625.1      ATGCAAAAACTAAAAATAACTGGCTTGTCATTGATCATATCCGGCCTGCTGATGGCACAG      60
DQ186611.1      ATGCAAAAACTAAAAATAACTGGCTTGTCATTGATCATATCCGGCCTGCTGATGGCACAG      60
GU229024.1      ATGCAAAAACTAAAAATAACTGGCTTGTCATTGATCATATCCGGCCTGCTGATGGCACAG      60
AF485769.1      ------------------------------------------------------------      0
EU254217.1      ------------------------------------------------------------      0
EU254232.1      ------------------------------------------------------------      0
EF450825.1      ------------------------------------------------------------      0
EF450824.1      ------------------------------------------------------------      0
KF955964.1      ------------------------------------------------------------      0

MF315078.1      ------------------------------------------------------------      0
HQ425626.1      GCTCATGCGGCAGAGCCCGTCTATCCAGACCAGCTGCGCCTGTTCTCACTGGGCCAGGAG      120
HQ425625.1      GCTCATGCGGCAGAGCCCGTCTATCCAGACCAGCTGCGCCTGTTCTCACTGGGCCAGGAG      120
DQ186611.1      GCTCATGCGGCAGAGCCCGTCTATCCAGACCAGCTGCGCCTGTTCTCACTGGGCCAGGAG      120
GU229024.1      GCTCATGCGGCAGAGCCCGTCTATCCAGACCAGCTGCGCCTGTTCTCACTGGGCCAGGAG      120
AF485769.1      ------------------------------------------------------------      0
EU254217.1      ------------------------------------------------------------      0
EU254232.1      ------------------------------------------------------------      0
EF450825.1      ---------GCAGAGCCCGTCTATCCAGACCAGCTTCGCCTGTTTTCACTGGGCCAGGAG      51
EF450824.1      ---------GCAGAGCCCGTCTATCCAGACCAGCTGCGCCTGTTCTCACTGGGCCAGGAG      51
KF955964.1      ------------------------------------------------------------      0

MF315078.1      ---------GACAAGTATCGCCCTATTACTCGAGAGGAAGCCCAGAGCGTTAAAAGCAAT      51
HQ425626.1      GTCTGTGGCGACAAGTATCGCCCTATTACTCGAGAGGAAGCCCAGAGCGTTAAAAGCAAT      180
HQ425625.1      GTCTGTGGCGACAAGTATCGCCCTATTACTCGAGAGGAAGCCCAGAGCGTTAAAAGCAAT      180
DQ186611.1      GTCTGTGGCGACAAGTATCGCCCTATTACTCGAGAGGAAGCCCAGAGCGTTAAAAGCAAT      180
GU229024.1      GTCTGTGGCGACAAGTATCGCCCTATTACTCGAGAGGAAGCCCAGAGCGTTAAAAGCAAT      180
AF485769.1      ------------------------------------------------------------      0
EU254217.1      ------------------------------------------------------------      0
EU254232.1      ------------------------------------------------------------      0
EF450825.1      GTATGTGGCGACAAGTATCACCCTGTTACTCGAGAAGAAGCCCAGAGCGTTAAAAGCAAA      111
EF450824.1      GTTTGTGGCGACAAGTATCGCCCTATTACTCGGGAAGAAGCCCAGAGCGTTAAAAGTAAT      111
KF955964.1      ------------------------------------------------------------      0
```

```
MF315078.1    ATTGTCAATATGATGGGGCAGTGGCAAATAAGCGGTATGGCCAACGGCTGGGTAATAATG    111
HQ425626.1    ATTGTCAATATGATGGGGCAGTGGCAAATAAGCGGTCTGGCCAACGGCTGGGTAATAATG    240
HQ425625.1    ATTGTCAATATGATGGGGCAGTGGCAAATAAGCGGTCTGGCCAACGGCTGGGTAATAATG    240
DQ186611.1    ATTGTCAATATGATGGGGCAGTGGCAAATAAGCGGTCTGGCCAACGGCTGGGTAATAATG    240
GU229024.1    ATTGTCAATATGATGGGGCAGTGGCAAATAAGCGGTCTGGCCAACGGCTGGGTAATAATG    240
AF485769.1    ------------------------------------------------------------    0
EU254217.1    ------------------------------------------------------------    0
EU254232.1    ------------------------------------------------------------    0
EF450825.1    ATTGTCGATATGATGGGACAGTGGCAAATAAGCGGTCTGGCCAACGGCTGGGTAATAATG    171
EF450824.1    ATTGTCAATATGATGGGGCAGTGGCAAATAAGCGGTCTGGCCAACGGCTGGGTAATAATG    171
KF955964.1    ------------------------------------------------------------    0

MF315078.1    GGGCCGGGTTATAATGGTGAAATAAAACCGGGCTCGGCGTCCAATACCTGGTGTTACCCG    171
HQ425626.1    GGGCCGGGTTATAATGGTGAAATAAAACCGGGCTCGGCGTCCAATACCTGGTGCTACCCG    300
HQ425625.1    GGGCCGGGTTATAATGGTGAAATAAAACCGGGCTCGGCGTCCAATACCTGGTGCTACCCG    300
DQ186611.1    GGGCCGGGTTATAATGGTGAAATAAAACCGGGCTCGGCGTCCAATACCTGGTGTTACCCG    300
GU229024.1    GGGCCGGGTTATAATGGTGAAATAAAACCGGGCTCGGCGTCCAATACCTGGTGTTACCCG    300
AF485769.1    ------------------------------------------------------------    0
EU254217.1    ------------------------------------------------------------    0
EU254232.1    ------------------------------------------------------------    0
EF450825.1    GGGCCGGGATATAATGGTGAAATTAAACCTGGCTCGGCGTCCAATACCTGGTGTTACCCG    231
EF450824.1    GGGCCGGGTTATAATGGTGAAATTAAACCGGGCTCGGCGTCCAATACCTGGTGTTACCCG    231
KF955964.1    ------------------------------------------------------------    0

MF315078.1    ATCAGTCCTGTTACCGGAGAAATACCAACCTTGTCACCGCTGGATATTCCAGACGGTGAC    231
HQ425626.1    ATCAATTCTGTTACCGGAGAAATACCAACCTTGTCAGCTCTGGATATTCCAGACGGTGAC    360
HQ425625.1    ATCAATTCTGTTACCGGAGAAATACCAACCTTGTCAGCTCTGGATATTCCAGACGGTGAC    360
DQ186611.1    ATCAATCCTGTTACCGGAGAAATACCAACCTTGTCAGCTCTGGATATTCCAGACGGTGAC    360
GU229024.1    ATCAGTCCTGTTACCGGAGAAATACCAACCTTGTCAGCTCTGGATATTCCAGACGGTGAC    360
AF485769.1    ------------------------------------------------------------    0
EU254217.1    ------------------------------------------------------------    0
EU254232.1    ------------------------------------------------------------    0
EF450825.1    GTCAATCCTGTCACCGGAGAAATACCAACCTTGTCAGCCCTGGATATTCCAGACGGTGAC    291
EF450824.1    GTCAATCCTGTTACCGGAGAAATACCAACCTTGTCAGCCCTGGATATTCCAGACGGTGAC    291
KF955964.1    ------------------------------------------------------------    0

MF315078.1    GAAGTGGACGTGCAGTGGCGACTGGTACACGACAGCGCGAATTTTATCAAGCCAACCAGC    291
HQ425626.1    GAAGTGGACGTGCAGTGGCGACTGGTACACGACAGCGCGAATTTTATCAAGCCAACCAGC    420
HQ425625.1    GAAGTGGACGTGCAGTGGCGACTGGTACACGACAGCGCGAATTTTATCAAGCCAACCAGC    420
DQ186611.1    GAAGTGGACGTGCAGTGGCGACTGGTACACGACAGCGCGAATTTTATCAAGCCAACCAGC    420
GU229024.1    GAAGTGGACGTGCAGTGGCGACTGGTACACGACAGCGCGAATTTTATCAAGCCAACCAGC    420
AF485769.1    ------------------------------------------------------------    0
EU254217.1    ------------------------------------------------------------    0
EU254232.1    ------------------------------------------------------------    0
EF450825.1    GAGGTGGACGTGCAGTGGCGACTGGTACACGACAGTGCGAATTTCATTAAGCCAACCAGC    351
EF450824.1    GAGGTGGACGTGCAGTGGCTACTGGTACACGACAGCGCGAATTTCATCAAGCCAGCCAGC    351
KF955964.1    ------------------------------------------------------------    0
```

```
MF315078.1    TATCTGGCGCATTATCTCGGTTATGCCTGGGTGGGTGGCAATCACAGCCAATATGTCGGT    351
HQ425626.1    TATCTGGCGCATTATCTCGGTTATGCCTGGGTGGGTGGCAATCACAGCCAATATGTCGGT    480
HQ425625.1    TATCTGGCGCATTATCTCGGTTATGCCTGGGTGGGTGGCAATCACAGCCAATATGTCGGT    480
DQ186611.1    TATCTGGCGCATTATCTCGGTTATGCCTGGGTGGGTGGCAATCACAGCCAATATGTCGGT    480
GU229024.1    TATCTGGCGCATTATCTCGGTTATGCCTGGGTGGGTGGCAATCACAGCCAATATGTCGGT    480
AF485769.1    ---------------------------------------AATCACAGCCAATATGTCGGT    21
EU254217.1    ------------------------------------------------------------    0
EU254232.1    ------------------------------------------------------------    0
EF450825.1    TATCTGGCGCATTATCTCGGTTATGCCTGGGTGGGTGGTAATCACAGCCAATATGTCGGT    411
EF450824.1    TATCTGGCGCATTATCTCGGTTATGCCTGGGTGGGCGGCAATCACAGCCAATATGTCGGT    411
KF955964.1    ------------------------------------------------------------    0

MF315078.1    GAAGACATGGACGTGACCCGTGATGGCGATGGCTGGGTGATCCGTGGCAACAATGACGGC    411
HQ425626.1    GAAGACATGGACGTGACCCGTGATGGCGATGGCTGGGTGATCCGTGGCAACAATGACGGC    540
HQ425625.1    GAAGACATGGACGTGACCCGTGATGGCGATGGCTGGGTGATCCGTGGCAACAATGACGGC    540
DQ186611.1    GAAGACATGGACGTGACCCGTGATGGCGATGGCTGGGTGATCCGTGGCAACAATGACGGC    540
GU229024.1    GAAGACATGGACGTGACCCGTGATGGCGATGGCTGGGTGATCCGTGGCAACAATGACGGC    540
AF485769.1    GAAGACATGGATGTGACCCGCGATGGCGATGGCTGGGTGATCCGTGGCAACAATGACGGC    81
EU254217.1    ----------------------------------------------------ATGACGGC    8
EU254232.1    ----------------------------------------------------ATGACGGC    8
EF450825.1    GAAGACATGGACGTGACCCGTGATGGCGATGGCTGGGTGATCCGTGGCAACAATGACGGC    471
EF450824.1    GAAGACATGGACGTGACCCGTGATGGCGATGGCTGGGTGATCCGTGGCAACAATGACGGC    471
KF955964.1    ------ATGGACGTGACCCGTGATGGCGATGGCTGGGTGATCCGTGGCAACAATGACGGC    54
                                                                  ********

MF315078.1    GGTTGCGAGGGGTATCGTTGTGGCGAGAAGACGGCCATCAAGGTCAGCAATTTTGCGTAC    471
HQ425626.1    GGTTGCGAGGGGTATCGTTGTGGCGAGAAGACGGCCATCAAGGTCAGCAATTTTGCGTAC    600
HQ425625.1    GGTTGCGAGAGGTATCGTTGCGGCGAGAAGACGGCCATCAAGGTCAGCAATTTTGCGTAC    600
DQ186611.1    GGTTGCGAGGGGTATCGTTGTGGCGAGAAGACGGCCATCAAGGTCAGCAATTTTGCGTAC    600
GU229024.1    GGTTGCGAGGGGTATCGTTGTGGCGAGAAGACGGCCATCAAGGTCAGCAATTTTGCGTAC    600
AF485769.1    GGTTGCGAGGGTTATCGTTGTGGCGAGAAGACGGCCATCAAGGTCAGCAATTTTGCGTAC    141
EU254217.1    GGTTGCGAGGGGTATCGTTGTGGCGAGAAGACGGCCATCAAGGTCAGCAACTTTGCATAC    68
EU254232.1    GGTTGCGAGGGGTATCGTTGTGGCGAGAAGACGGCCATCAAGGTCAGCAACTTTGCATAC    68
EF450825.1    GGTTGCGAGGGGTATCGTTGTGGGGAGAAGACGGCCATCAAGGTCAGCAATTTTGCGTAC    531
EF450824.1    GGTTGCGAGGGGTATCGTTGTGGCGAGAAGACGGCCATCAAGGTCAGCAACTTTGCGTAC    531
KF955964.1    GGTTGCGAGGGTTATCGTTGTGGCGAGAAGACGGCCATCAAGGTCAGCAATTTTGCGTAC    114
              ********* * ******** ** ************************** ***** ***

MF315078.1    AACCTGGACCCTGACAGCTTCAAACATGGTGATGTGACCCAGAAGGATCGCCAGCTGGTC    531
HQ425626.1    AACCTGGACCCTGACAGCTTCAAACATGGTGATGTGACCCAGTCTGATCGCCAGCTGGTC    660
HQ425625.1    AACCTGGACCCTGACAGCTTCAAACATGGTGATGTGACCCAGTCTGATCGCCAGCTGGTC    660
DQ186611.1    AACCTGGACCCTGACAGCTTCAAACATGGTGATGTGACCCAGTCTGATCGCCAGCTGGTC    660
GU229024.1    AACCTGGACCCTGACAGCTTCAAACATGGTGATGTGACCCAGTCTGATCGCCAGCTGGTC    660
AF485769.1    AACCTGGACCCTGACAGCTTCAAACATGGTGACGTGACCCAGTCTGACCGTCAGCTGGTC    201
EU254217.1    AACCTGGACCCTGACAGTTTCAAACATGGTGACGTGACCCAGTCTGATCGCCAGCTGGTC    128
EU254232.1    AACCTGGACCCTGACAGTTTCAAACATGGTGACGTGACCCAGTCTGATCGCCAGCTGGTC    128
EF450825.1    AACCTGGACCCTGACAGCTTCAAACATGGTGACGTGACCCAGTCTGATCGCCAACTGGTC    591
EF450824.1    AACCTGGACCCTGACAGTTTCAAACATGGTGACGTGACCCAGTCTGATCGCCAGCTGGTT    591
KF955964.1    AACCTGGACCCTGACAGTTTCAAACATGGTGACGTGACCCAGTCTGATCGCCAACTGGTC    174
              ***************** ************** *********   ** ** ** *****
```

```
MF315078.1     AAGACGGTGGTGGGCTGGGCGATCAACGACAGCGACACCCCGCAATCCGGCTATGATGTC     591
HQ425626.1     AAGACGGTGGTGGGCTGGGCGATCAACGACAGCGACACCCCGCAATCCGGCTATGATGTC     720
HQ425625.1     AAGACGGTGGTGGGCTGGGCGATCAACGACAGCGACACCCCGCAATCCGGCTATGATGTC     720
DQ186611.1     AAGACGGTGGTGGGCTGGGCGATCAACGACAGCGACACCCCGCAATCCGGCTATGATGTC     720
GU229024.1     AAGACGGTGGTGGGCTGGGCGATCAACGACAGCGACACCCCGCAATCCGGCTATGATGTC     720
AF485769.1     AAGACGGTGGTGGGCTGGGCGATCAACGACAGCGACACCCCGCAATCCGGGTATGACGTC     261
EU254217.1     AAGACGGTGGTGGGCTGGGCGATCAACGACAGCGACACCCCGCAATCCGGTTATGATGTC     188
EU254232.1     AAGACGGTGGTGGGCTGGGCGATCAACGACAGCGACACCCCGCAATCCGGTTATGATGTC     188
EF450825.1     AAGACGGTGGTGGGCTGGGCGATCAACGACAGCGACACCCCGCAATCCGGCTATGATGTC     651
EF450824.1     AAGACGGTGGTGGGCTGGGCGATCAACGACAGCGACACCCCGCAATCCGGCTATGATGTC     651
KF955964.1     AAGACGGTGGTGGGCTGGGCGATCAACGACAGCGACACCCCGCAATCCGGTTATGATGTC     234
               ************************************************** ***** ***

MF315078.1     ACCCTGCGTTACGATACCGCCACCAACTGGTCCAAGACCAATACCTATGGCCTGAGCGAG     651
HQ425626.1     ACCCTGCGTTACGATACCGCCACCAACTGGTCCAAGACCAATACCTATGGCCTGAGCGAG     780
HQ425625.1     ACCCTGCGTTACGATACCGCCACCAACTGGTCCAAGACCAATACCTATGGCCTGAGCGAG     780
DQ186611.1     ACCCTGCGTTACGATACCGCCACCAACTGGTCCAAGACCAATACCTATGGCCTGAGCGAG     780
GU229024.1     ACCCTGCGTTACGATACCGCCACCAACTGGTCCAAGACCAATACCTATGGCCTGAGCGAG     780
AF485769.1     ACTCTGCGTTACGATACTGCCACCAACTGGTCCAAGACCAATACCTATGGCCTGAGCGAG     321
EU254217.1     ACCCTACGTTACGATACTGCCACCAACTGGTCCAAGACCAATACCTATGGCCTGAGCGAG     248
EU254232.1     ACCCTACGTTACGATACTGCCACCAACTGGTCCATGAGCAATACCTATGGCCTGAGCGAG     248
EF450825.1     ACCCTGCGTTACGATACTGCCACCAACTGGTCCAAGACCAATACCTATGGCCTGAGCGAG     711
EF450824.1     ACCCTGCGTTACGATACTGCCACCAACTGGTCCAAGACCAATACCTATGGCCTGAGCGAG     711
KF955964.1     ACCCTGCGTTACGATACCGCCACCAACTGGTCCAAGACCAATACCTATGGCCTGAGCGAG     294
               ** ** *********** **************** ** **********************

MF315078.1     AAGGTGACCACCAAGAACAAGTTCAAGTGGCCACTGGTAGGGGAAACCGAACTCTCCATC     711
HQ425626.1     AAGGTGACCACCAAGAACAAGTTCAAGTGGCCACTGGTAGGGGAAACCGAACTCTCCATC     840
HQ425625.1     AAGGTGACCACCAAGAACAAGTTCAAGTGGCCGCTGGTAGGGGAAACCGAACTCTCTATC     840
DQ186611.1     AAGGTGACCACCAAGAACAAGTTCAAGTGGCCACTGGTAGGGGAAACCGAACTCTCCATC     840
GU229024.1     AAGGTGACCACCAAGAACAAGTTCAAGTGGCCACTGGTAGGGGAAACCGAACTCTCCATC     840
AF485769.1     AAGGTGACCACCAAGAACAAGTTCAAGTGGCCACTGTAGGGGAAACCGAACTCTCCATC      381
EU254217.1     AAGGTGACCACCAAGAACAAGTTCAAGTGGCCACTGGTAGGAGAAACCGAACTCTCCATC     308
EU254232.1     AAGGTGACCACCAAGAACAAGTTCAAGTGGCCACTGGTAGGAGAAACCGAACTCTCCATC     308
EF450825.1     AAGGTGACCACCAAGAACAAGTTCAAGTGGCCACTGGTAGGGGAAACCGAGCTCTCCATC     771
EF450824.1     AAGGTGACCACCAAGAACAAGTTCAAGTGGCCACTGGTAGGGGAAACCGAGCTCTCCATC     771
KF955964.1     AAGGTGACCACCAAGAACAAGTTCAAGTGGCCACTGGTAGGGGAAACCGAACTCTCCATC     354
               ******************************** ******** ******** ***** ***

MF315078.1     GAGATTGCGGCCAAGCAGTCCTGGGCCTCCCAGAACGGGGGAGCTACCACCGCCTCCCTG     771
HQ425626.1     GAGATTGTGGCCAACCAGTCCTGGGCCTCCCAGAACGGGGGATCTACCACCACCTCCCTG     900
HQ425625.1     GAGATTGTGGCCAACCAGTCCTGGGCCTCCCAGAACGGGGGATCTACCACCACCTCCCTG     900
DQ186611.1     GAGATTGCGGCCAACCAGTCCTGGGCCTCCCAGAACGGGGGATCTACCACCACCTCCCTG     900
GU229024.1     GAGATTGCGGCCAACCAGTCCTGGGCCTCCCAGAACGGGGGAGCTACCACCGCCTCCCTG     900
AF485769.1     GAGATTGCGGCCAACCAGTCATGGGCATCCCAGAACGGGGGTTCTACCACCACCTCCCTG     441
EU254217.1     GAGATTGCGGCCAACCAGTCCTGGGCATCCCAGAACGGGGGCTCTACCACCACCTCCCTG     368
EU254232.1     GAGATTGCGGCCAACCAGATCTCGGCATCCCAGAACGGGGGCTCTACCACCACCTCCCTG     368
EF450825.1     GAGATTGTTGCCAACCAGTCCTGGGCCTCCCAGAACGGGGGCTCTACCACCACCTCTCTG     831
EF450824.1     GAGATTGCGGCCAACCAGTCCTGGGCATCCCAGAACGGGGGCTCTACCACCACCTCCCTG     831
KF955964.1     GAGATTGCGGCCAACCAGTCCTGGGCATCCCAGAACGGGGGCTCTACCACCACCTCCCTG     414
               *******  ***** ***   * *** *************  ******** **** ***
```

```
MF315078.1      TCGCAATCCGTGCGGCCGACGGTGCCGGCCCGCTCCGAGATCCCGGTGAACATCGAGCTC      831
HQ425626.1      TCGCAATCCGTGCGGCCGACGGTGCCGGCCCGCTCCAAGATCCCGGTGAAGATCGAGCTC      960
HQ425625.1      TCGCAATCCGTGCGGCCGACGGTGCCGGCCCGCTCCAAGATCCCGGTGAAGATCGAGCTC      960
DQ186611.1      TCGCAATCCGTGCGGTCGACGGTGCCGGCCCGCTCCAAGATCCCGGTGAAGATCGAGCTC      960
GU229024.1      TCGCAATCCGTGCGGCCGACGGTGCCGGCCCGCTCCAAGATCCCGGTGAAGATCGAGCTC      960
AF485769.1      TCGCAATCCGTGCGGCCGACGGTGCCGGCCCGCTCCAAGATCCCGGTGAAGATCGAGCTC      501
EU254217.1      TCGCAATCCGTGCGGCCAACTGTGCCGGCCCGCTCCAAGATCCCGGTGAAGATCGAGCTC      428
EU254232.1      TCGCAATCCGTGCGGCCAACTGTGCCGGCCCGCTCCAAGATCCCGGTGAAGATCGAGCTC      428
EF450825.1      TCGCAATCCGTGCGGCCAACTGTGCCGGCCCGCTCCAAGATCCCGGTGAAGATCGAGCTC      891
EF450824.1      TCGCAATCCGTGCGGCCGACGGTGCCGGCCCGCTCCAAGATCCCGGTGAAGATCGAGCTC      891
KF955964.1      TCGCAATCCGTGCGGCCGACAGTGCCGGCCCGCTCCAAGATCCCGGTGAAGATCGAGCTC      474
                ************** * ** ************** ************* *********

MF315078.1      TACAAGGCTGACGTGTCCTATCCCTATGAATTCAAAGCCGATGTCAGCTATGACCTGACC      891
HQ425626.1      TACAAGGCTGACATCTCCTATCCCTATGAATTCAAAGCCGATGTCAGCTATGACCTGACC      1020
HQ425625.1      TACAAGGCTGACATCTCCTATCCCTATGAATTCAAAGCCGATGTCAGCTATGACCTGACC      1020
DQ186611.1      TACAAGGCTGACATCTCCTATCCCTATGAATTCAAAGCCGATGTCAGCTATGACCTGACC      1020
GU229024.1      TACAAGGCTGACATCTCCTATCCCTATGAATTCAAAGCCGATGTCAGCTATGACCTGACC      1020
AF485769.1      TACAAGGCTGATATCTCCTATCCCTATGAGTTCAAGGCCGATGTCAGTTATGACCTGACT      561
EU254217.1      TACAAGGCTGACATCTCCTATCCCTATGAATTCAAGGCCGATGTCAGCTATGACCTGACG      488
EU254232.1      TACAAGGCTGACATCTCCTATCCCTATGAATTCAAGGCCGATGTCAGCTATGACCTGACG      488
EF450825.1      TACAAGGCTGACATCTCCTATCCCTATGAGTTCAAGGCCGATGTCAGCTATGACCTGACC      951
EF450824.1      TACAAGGCTGACATCTCCTATCCCTATGAATTCAAGGCCGATGTCAGCTATGACCTGACA      951
KF955964.1      TACAAGGCTGACATCTCCTATCCCTATGAATTCAAGGCCGATGTCAGCTATGACCTGACC      534
                ***********  * ************** ***** *********** ***********
MF315078.1      CTGAGCGGCTTCCTGCGCTGGGGCGGCAATGCCTGGTATACCCATCCGGACAATCGCCCG      951
HQ425626.1      CTGAGCGGCTTCCTGCGCTGGGGCGGCAATGCCTGGTATACCCATCCGGACAACCGCCCG      1080
HQ425625.1      CTGAGCGGCTTCCTGCGCTGGGGCGGCAATGCCTGGTATACCCATCCGGACAACCGCCCG      1080
DQ186611.1      CTGAGCGGTTTCCTGCGCTGGGGCGGCAATGCCTGGTATACCCATCCGGACAACCGCCCG      1080
GU229024.1      CTGAGCGGCTTCCTGCGCTGGGGCGGCAATGCCTGGTATACCCATCCGGACAACCGCCCG      1080
AF485769.1      CTGAGTGGCTTCCTGCGTTGGGGTGGCAATGCCTGGTATGCCCATCCGGACAATCGCCCG      621
EU254217.1      CTGAGTGGCTTCCTGCGCTGGGGCGGCAATGCCTGGTATACCCATCCGGACAACCGCCCG      548
EU254232.1      CTGAGTGGCTTCCTGCGCTGGGGCGGCAATGCCTGGTATACCCATCCGGACAACCGCCCG      548
EF450825.1      CTGAGCGGCTTCCTGCGCTGGGGCGGCAATGCCTGGTATACCCATCCGGACAACCGCCCG      1011
EF450824.1      CTGAGTGGCTTCCTGCGCTGGGGCGGCAATGCCTGGTATACCCATCCGGACAACCGCCCG      1011
KF955964.1      CTGAGCGGCTTCCTGCGCTGGGGCGGCAATGCCTGGTATACCCATCCGGACAACCGCCCG      594
                ***** ** ******** ***** *************** ************* ******
MF315078.1      AACTGGAACCACACCTTCGTCATCGGGCCGTACAAGGACAAGGCGAGCAGCATTCGTTAC      1011
HQ425626.1      AACTGGAACCACACCTTCGTCATCGGGCCGTACAAGGACAAGGCGAGCAGCATTCGTTAC      1140
HQ425625.1      AACTGGAACCACACCTTCGTCATCGGGCCGTACAAGGACAAGGCGAGCAGCATTCGTTAC      1140
DQ186611.1      AACTGGAACCACACCTTCGTCATCGGGCCGTACAAGGACAAGGCGAGCAGCATTCGTTAC      1140
GU229024.1      AACTGGAACCACACCTTCGTCATCGGGCCGTACAAGGACAAGGCGAGCAGCATTCGTTAC      1140
AF485769.1      AACTGGAACCACACCTTCGTTATCGGGCCGTACAAGGACAAGGCGAGCAGTATTCGCTAC      681
EU254217.1      AACTGGAACCACA-----------------------------------------------      561
EU254232.1      CACTGGAACCACA-----------------------------------------------      561
EF450825.1      AACTGGAGCCACACCTTCGTCATCGGGCCGTACAAGGACAAGGCGAGCAGCATTCGCTAC      1071
EF450824.1      AACTGGAACCACACCTTCGTCATCGGGCCGTACAAGGACAAGGCGAGCAGCATTCGCTAC      1071
KF955964.1      AACTGGAACCACACCTTCGTCATCGGGCCGTACAAGGACAAGGCGAGCAGCATTCGCTAC      654
                 ****** *****
```

```
MF315078.1      CAGTGGGACAAGCGCTATATCACCGGTGAACTGAACTGGTGGGACTGGAACTGGACCATA    1071
HQ425626.1      CAGTGGGACAAGCGCTATATCCCGGGTGAAGTGAAGTGGTGGGACTGGAACTGGACCATA    1200
HQ425625.1      CAGTGGGACAAGCGCTATATCCCGGGTGAAGTGAAGTGGTGGGACTGGAACTGGACCATA    1200
DQ186611.1      CAGTGGGACAAGCGCTATATCCCGGGTGAAGTGAAGTGGTGGGACTGGAACTGGACCATA    1200
GU229024.1      CAGTGGGACAAGCGCTATATCCCGGGTGAAGTGAAGTGGTGGGACTGGAACTGGACCATA    1200
AF485769.1      CAGTGGGACAAGCGTTACATCCCGGGTGAAGTGAAGTGGTGGGACTGGAACTGGACCATC    741
EU254217.1      ------------------------------------------------------------    561
EU254232.1      ------------------------------------------------------------    561
EF450825.1      CAGTGGGACAAGCGTTACATCCCGGGTGAAGTGAAGTGGTGGGACTGGAACTGGACCATC    1131
EF450824.1      CAGTGGGACAAGCGTTACATCCCGGGTGAAGTGAAGTGGTGGGACTGGAACTGGACCATC    1131
KF955964.1      CAGTGGGACAAGCGTTACATCCCGGGTGAAGTGAAGTGGT--------------------    694

MF315078.1      CAGCAGAACGGCCTGTCTACCATGCAGAAC------------------------------    1101
HQ425626.1      CAGCAGAACGGCCTGTCTACCATGCAGAACAATCTGGCCAGAGTGCTGCGCCCGGTGCGG    1260
HQ425625.1      CAGCAGAACGGCCTGTCTACCATGCAGAACAATCTGGCCAGAGTGCTGCGCCCGGTGCGG    1260
DQ186611.1      CAGCAGAACGGCCTGTCTACCATGCAGAACAATCTGGCCAGAGTGCTGCGCCCGGTGCGG    1260
GU229024.1      CAGCAGAACGGCCTGTCTACCATGCAGAACAATCTGGCCAGAGTGCTGCGCCCGGTGCGG    1260
AF485769.1      CAGCAGAACGGCCTGTCTACCATGCAGAACAACCTGGCCAGAGTGCTGCGCCCGGTGCGG    801
EU254217.1      ------------------------------------------------------------    561
EU254232.1      ------------------------------------------------------------    561
EF450825.1      CAGCAGAACGGCCTGTCTACCATGCAGAACAACCTGGCCAGAGTGCTGCGCCCGGTGCGG    1191
EF450824.1      CAGCAGAACGGCCTGTCTACCATGCAGAACAACCTGGCCAGAGTGCTGCGCCCGGTGCGG    1191
KF955964.1      ------------------------------------------------------------    694

MF315078.1      ------------------------------------------------------------    1101
HQ425626.1      GTGGGGATCACCGGCGATTTCAGTGCCGAGAGCCAGTTTGCCGGCAACATCGAGATCGGT    1320
HQ425625.1      GTGGGGATCACCGGCGATTTCAGTGCCGAGAGCCAGTTTGCCGGCAACATCGAGATCGGT    1320
DQ186611.1      GCGGGGATCACCGGCGATTTCAGTGCCGAGAGCCAGTTTGCCGGCAACATCGAGATCGGT    1320
GU229024.1      GCGGGGATCACCGGCGATTTCAGTGCCGAGAGCCAGTTTGCCGGCAACATCGAGATC---    1317
AF485769.1      GCGGGGATCACCGGCGATTTCAGCGCCGAGAGCCAGTTTGCTGGCAATATCGAAATCGGT    861
EU254217.1      ------------------------------------------------------------    561
EU254232.1      ------------------------------------------------------------    561
EF450825.1      GCGGGGATCACCGGCGATTTCAGTGCTGAGAGCCAGTTTGCCGGCAACATCGAGATCGGT    1251
EF450824.1      GCGGGGATCACCGGCGATTTCAGTGCTGAGAGCCAGTTTGCCGGCAACATCGAGATCGGT    1251
KF955964.1      ------------------------------------------------------------    694

MF315078.1      ------------------------------------------------------------    1101
HQ425626.1      GCTCCGGTGCCGCTCGCGGCAGACAGCAAGGTGCGTCGTACCCGCAGCGTGGACGGCGCT    1380
HQ425625.1      GCTCCGGTGCCGCTCGCGGCAGACAGCAAGGTGCGTCGTACCCGCAGCGTGGACGGCGCT    1380
DQ186611.1      GCTCCGGTGCCGCTCGCGGCAGACAGCAAGGTGCGTCGTACCCGCAGCGTGGACGGCGCT    1380
GU229024.1      ------------------------------------------------------------    1317
AF485769.1      GCGCCCGTGCCCCTCGCGGCGGATGGTAAGGCGCCTCGAGCTCTCAGTGCGAGAGGAGGA    921
EU254217.1      ------------------------------------------------------------    561
EU254232.1      ------------------------------------------------------------    561
EF450825.1      GCTCCGGTGCCGCTCGCGGCAGACAGCAAGGTGCGTCGTACCCGCAGCGTGGACGGCGCT    1311
EF450824.1      GCTCCGGTGCCGCTCGCGGCAGACAGCAAGGTGCGTCGTACCCGCAGCGTGGACGGCGCT    1311
KF955964.1      ------------------------------------------------------------    694
```

```
MF315078.1    ------------------------------------------------------------    1101
HQ425626.1    GGTCAGGGCCTGCGGCTGGAGATCCCGCTCGATGCACAAGAGCTCTCCGGGCTTGGCTTC    1440
HQ425625.1    GGTCAGGGCCTGCGGCTGGAGATCCCGCTCGATGCACAAGAGCTCTCCGGGCTTGGCTTC    1440
DQ186611.1    GGTCAGGGCCTGCGGCTGGAGATCCCGCTCGATGCACAAGAGCTCTCCGGGCTTGGCTTC    1440
GU229024.1    ------------------------------------------------------------    1317
AF485769.1    GAACAGGGCCTGCGACTGGAGATCCCGCTCGATGCGCAAGAGCTCTCCGGGCTTGGCTTC    981
EU254217.1    ------------------------------------------------------------    561
EU254232.1    ------------------------------------------------------------    561
EF450825.1    GGTCAGGGCCTGAGGCTGGAGATCCCGCTCGATGCGCAAGAGCTCTCCGGGCTTGGCTTC    1371
EF450824.1    GGTCAGGGCCTGAGGCTGGAGATCCCGCTCGATGCGCAAGAGCTCTCCGGGCTTGGCTTC    1371
KF955964.1    ------------------------------------------------------------    694

MF315078.1    ------------------------------------------1101
HQ425626.1    AGCAACGTCAGCCTGAGCGTGACGCCAGTTGCCAATCAATAA1482
HQ425625.1    AGCAACGTCAGCCTGAGCGTGACGCCAGTTGCCAATCAATAA1482
DQ186611.1    AGCAACGTCAGCCTGAGCGTGACGCCAGTTGCCAATCAATAA1482
GU229024.1    ------------------------------------------1317
AF485769.1    AGCAACGTCAGCCTGAGCGTG---------------------1002
EU254217.1    ------------------------------------------561
EU254232.1    ------------------------------------------561
EF450825.1    AACAACGTCAGCCTCAGCGTGA--------------------1393
EF450824.1    AACAACGTCAGCCTCAGCGTGA--------------------1393
KF955964.1    ------------------------------------------694
```

Figure S4. The aligned results and the conserved regions of *A. hydrophila aerA* gene. The sequences highlighted with green and yellow were primer AF5 and AR5, respectively. The sequences with the red underline and blue underline were used to design crRNA ACR1 and ACR2, respectively. *: indicates that the nucleotide is conserved among these sequences.

To design RAA primers and crRNA of *hlyA* gene, several sequences of *A. hydrophila hlyA* obtained from GenBank were also aligned using the online tool, Clustal Omega. The results were shown in below (**Figure S5**). To improve the specificity of our developed method, dRAA-CRISPR/Cas12a, we designed two crRNA sequences (HCR1 and HCR2) targeting *hlyA* gene which were not conserved in other species of *Aeromonas*.

```
AY442273.1      ATGAAAAACAAAAAACCACGCAAATTCATCACGCAAGCCCCCACTCTTAGTCTGCTCGCG      60
GU229025.1      ATGAAAAACAAAAAACCACGCAAATTCATCACGCAAGCCCCCACTCTTAGTCTGCTCGCG      60
LR963141.1      ATGAAAAACAAAAAACCACGCAAATTCATCACGCAAGCCCCCACTCTTAGTCTGCTCGCG      60
KC812121.1      ATGAAAAACAAAAAACCACGCAAATTCATCACACAAGCCCCCACTCTCAGTCTGCTCACG      60
KC812120.1      ATGAAAAACAAAAAACCACGCAAATTCATCACGCAAGCCCCCACTCTCAGTCTGCTCGCG      60
U81555.1        ATGAAAAACAAAAAACCACGCAAATTCATCACGCAAGCCCCCACTCTCAGTCTGCTCGCG      60
KF786302.1      ATGAAAAACAAAAAACCACGCAAATTCATCACGCAAGCCCCCACTCTCAGTCTGCTCGCG      60
KC812122.1      ATGAAAAACAAAAAACCACGCAAATTCATCACGCAAGCCCCCACTCTCAGTCTGCTCGCG      60
                ******************************** ************** ********* **

AY442273.1      CTGGCCTTGTTGGCAGGCAGCGTGCAAGCCGAAGATATTGGCGAACGTACCGACCAGGGC      120
GU229025.1      CTGGCCTTGTTGGCAGGCAGCGTGCAAGCCGAAGATATTGGCGAACGTACCGACCAGGGC      120
LR963141.1      CTGGCCTTGTTGGCAGGCAGCGTGCAAGCCGAAGATATTGGCGAACGTACCGACCAGGGC      120
KC812121.1      CTGGCCTTGTTGGCAGGCAGCGTGCAAGCCGAAGATATTGGCGAACGTACCGACCAGGGC      120
KC812120.1      CTGGCCTTGTTGGCAGGCAGCGTGCAAGCCGAAGATATTGGCGAACGTACCGACCAGGGC      120
U81555.1        CTGGCCTTGTTGGCAGGCAGCGTGCATGCCGAAGATATTGGCGAACGTACCGACCAGGGC      120
KF786302.1      CTGGCCTTGTTGGCAGGCAGCGTGCATGCCGAAGATATTGGCGAACGTACCGACCAGGGC      120
KC812122.1      CTGGCCTTGTTGGCAGGCAGCGTGCATGCCGAAGATATTGGCGAACGTACCGACCAGGGC      120
                ************************** *********************************

AY442273.1      ACCGCCATGCTGGCCAGCCTGCAATCCGAACAGGGTCTGATTTACCTCAATGCCGACGTC      180
GU229025.1      ACCGCCATGCTGGCCAGCCTGCAATCCGAACAGGGTCTGATTTACCTCAATGCCGACGTC      180
LR963141.1      ACCGCCATGCTGGCCAGCCTGCAATCCGAACAGGGTCTGATTTACCTCAATGCCGACGTC      180
KC812121.1      ACCGCCATGCTGGCCAGCCTGCAATCCGAACAGGGTCTGATTTACCTCAATGCCGACGTC      180
KC812120.1      ACCGCCATGCTGGCCAGCCTGCAATCCGAACAGGGTCTGATTTACCTCAATGCCGACGTC      180
U81555.1        ACCACCATGCTGGCCAGCCTGCAATCCGAACAGGGTCTGATTTACCTCAATGCCGACGTC      180
KF786302.1      ACCGCCATGCTGGCCAGCCTGCAATCCGAACAGGGTCTGATTTACCTCAATGCCGACGTC      180
KC812122.1      ACCGCCATGCTGGCCAGCCTGCAATCCGAACAGGGTCTGATTTACCTCAATGCCGACGTC      180
                *** ********************************************************
```

```
AY442273.1      TGGCTGAAGGGGCAGGGGGCGACGCCGCTCATGACCCGGGATCAGCTGCGCGAGCGGGTG      240
GU229025.1      TGGCTGAAGGGGCAGGGGGCGACGCCGCTCATGACCCGGGATCAGCTGCGCGAGCGGGTG      240
LR963141.1      TGGCTGAAGGGGCAGGGGGCGACGCCGCTCATGACCCGGGATCAGCTGCGCGAGCGGGTG      240
KC812121.1      TGGCTGAAGGGGCAGGGGGCGACGCCGCTCATGACCCGGGATCAGCTGCGCGAGCGGGTG      240
KC812120.1      TGGTTGAAGGGGCAGGGGGCGACGCCGCCCATGACCCGGGATCAGCTGCGCGAGCGGGTG      240
U81555.1        TGGCTGAAGGGGCAGGGGGCGACGCCGCTCATGACCCGGGATCAGCTGCGCGAGCGGGTG      240
KF786302.1      TGGCTGAAGGGGCAGGGGGCGACGCCGCTCATGACCCGGGATCAGCTGCGCGAGCGGGTG      240
KC812122.1      TGGCTGAAGGGGCAGGGGGCGACGCCGCTCATGACCCGGGATCAGCTGCGCGAGCGGGTG      240
                *** ********************** *********************************

AY442273.1      CTGGCACGGGGCGAGCGTCTGTTCATCGATTTCAGCGCCGTCACCGACAAGAATGAGCGG      300
GU229025.1      CTGGCACGGGGCGAGCGTCTGTTCATCGATTTCAGCGCCGTCACCGACAAGAATGAGCGG      300
LR963141.1      CTGGCACGGGGCGAGCGTCTGTTCATCGATTTCAGCGCCGTCACCGACAAGAATGAGCGG      300
KC812121.1      CTGGCACGGGGCGAGCGTCTGTTCATCGATTTCAGCGCCGTCACCGACAAGAATGAGCGA      300
KC812120.1      CTGGCACGGGGCGAGCGTCTGTTCATCGATTTCAGCGCCGTCACCGACAAGAATGAGCGG      300
U81555.1        CTGGAACGGGGCGAGCGTCTGTTCATCGATCTCAGCGCCGTCACCGACAAGAATGAGCGG      300
KF786302.1      CTGGAACGGGGCGAGCGTCTGTTCATCGATTTCAGCGCCGTCACCGACAAGAATGAGCGA      300
KC812122.1      CTGGAACGGGGCGAGCGTCTGTTCATCGATTTCAGCGCCGTCACCGACAAGAATGAGCGA      300
                **** ************************* **************************** 


AY442273.1      CAGCAGGCCAGAAAGGCCATGGAGCAGCTGGCCGGCATCTCTTTTGATGCGGACTGGGTG      360
GU229025.1      CAGCAGGCCAGAAAGGCCATGGAGCAGCTGGCCGGCATCTCTTTTGATGCGGACTGGGTG      360
LR963141.1      CAGCAGGCCAGAAAGGCCATGGAGCAGCTGGCCGGCATCTCTTTTGATGCGGACTGGGTG      360
KC812121.1      CAGCAGGCCAGAAAGGCCATGGAGCAGCTGGCCGGCATCTCTTTTGATGCGGACTGGGTG      360
KC812120.1      CAGCAGGCCAGAAAGGCCATGGAGCAGCTGGCCGGCATCTCTTTTGATGCAGACTGGGTG      360
U81555.1        CAGCAAGCCAGAAAAGCCATGGAGCAGCTGGCCGGCATCTCTTTTGATGCAGACTGGGTG      360
KF786302.1      CAGCAAGCCAGAAAAGCCATGGAGCAGCTGGCCGGCATCTCTTTTGATGCAGACTGGGTG      360
KC812122.1      CAGCAAGCCAGAAAAGCCATGGAGCAGCTGGCCGGCATCTCTTTTGATGCAGACTGGGTG      360
                ***** ******** ************************************ ********

AY442273.1      CTGGTGTCCGGCTACAAGGGGGAGTTGCTGTTCACCCCGCTGGGAGGCGTCGATGACCCG      420
GU229025.1      CTGGTGTCCGGCTACAAGGGGGAGTTGCTGTTCACCCCGCTGGGAGGCGTCGATGACCCG      420
LR963141.1      CTGGTGTCCGGCTACAAGGGGGAGTTGCTGTTCACCCCGCTGGGAGGCGTCGATGACCCG      420
KC812121.1      CTGGTGTCCGGCTACAAGGGGGAGCTGCTGTTCACCCCGCTGGGAGGCGTCGATGACCCG      420
KC812120.1      CTGGTGTCCGGCTACAAGGGGGAGCTGCTGTTCACCCCGCTGGGAGGCGTCGATGACCCG      420
U81555.1        CTGGTGTCAGGCTACAAGGGGGAGCTGCTGTTCACCCCGCTGGGGGGCGTCGATGACCCG      420
KF786302.1      CTGGTGTCAGGCTACAAGGGGGAGCTGCTGTTCACCCCGCTGGGGGGCGTCGATGACCCG      420
KC812122.1      CTGGTGTCAGGCTACAAGGGGGAGCTGCTGTTCACCCCGCTGGGGGGCGTCGATGACCCG      420
                ******** *************** ******************* ***************

AY442273.1      GCCTTCTATCAGGTGATGGAGCGGGTCGAGAGCCTGGAAGGGCAGGGCAAGCGCCACAAG      480
GU229025.1      TCCTTCTATCAGGTGATGGAGCGGGTCGAGAGCCTGGAAGGGCAGGGCAAGCGCCACAAG      480
LR963141.1      GCCTTCTATCAGGTGATGGAGCGGGTCGAGAGCCTGGAAGGGCAGGGCAAGCGCCACAAG      480
KC812121.1      GCCTTCTATCAGGTAATGGAGCGGGTCGAGAGCCTGGAAGGGCAGGGCAAGCGCCACAAG      480
KC812120.1      GCCTTCTATCAGGTGATGGAGCGGGTCGAGAGCCTGGATGGGCAGGGCAAGCGCCACAAG      480
U81555.1        GCCTTCTATCAGGTGATGGAGCGGGTCGAGAGCCTGGAAGGGCAGGGCAAGCGCCACAAG      480
KF786302.1      GCCTTCTATCAGGTGATGGAGCGGGTCGAGAGCCTGGAAGGGCAGGGCAAGCGCCACAAG      480
KC812122.1      GCCTTCTATCAGGTGATGGAGCGGGTCGAGAGCCTGGAAGGGCAGGGCAAGCGCCACAAG      480
                 ************* *********************** **********************
```

```
AY442273.1      CGCTCGCTGACCCAGCCGCCTGCCGCCGAGGCCGGCCTGCCCCACGTGGCCTTCTACCTC      540
GU229025.1      CGCTCGCTGACCCAGCCGCCTGCCGCCGAGGCCGGCCTGCCCCACGTGGCCTTCTACCTC      540
LR963141.1      CGCTCGCTGACCCAGCCGCCTGCCGCCGAGGCCGGCCTGCCCCACGTGGCCTTCTACCTC      540
KC812121.1      CGCTCGCTGACCCAGCCGCCCGCCGCCGAGGCTGGCTTGCCCCACGTGGCCTTCTACCTC      540
KC812120.1      CGCTCGCTGACCCAGCCGCCTGCCGCCGAGGCCGGCCTGCCCCACGTGGCCTTCTACCTC      540
U81555.1        CGCTCGCTGACCCAGCCGCCGCCCGCCGAGACCGGTCTGCCCCACGTGGCCTTCTACCTC      540
KF786302.1      CGCTCGCTGACCCAGCCGCCCGCCGCCGAGGCCGGTCTGCCCCACGTGGCCTTCTACCTC      540
KC812122.1      CGCTCGCTGACCCAGCCGCCCGCCGCCGAGGCCGGTCTGCCCCACGTGGCCTTCTACCTC      540
                ********************  ******** * * **  ***********************

AY442273.1      AACGTCAACCGCAAGATCAGCGATGCCGAGTGCACCTTCCCGCGCTCGCGCACCTGGAGC      600
GU229025.1      AACGTCAACCGCAAGATCAGCGATGCCGAGTGCACCTTCCCGCGCTCGCGCACCTGGAGC      600
LR963141.1      AACGTCAACCGCAAGATCAGCGATGCCGAGTGCACCTTCCCGCGCTCGCGCACCTGGAGC      600
KC812121.1      AACGTCAACCGCAAGATCAGCGATGCCGAGTGTACCTTCCCGCGCTCGCGCACCTGGAGC      600
KC812120.1      AACGTCAACCGCAAGATCAGCGATGCCGAGTGCACCTTCCCGCGCTCGCGCACCTGGAGC      600
U81555.1        AACGTCAACCGCAAGATCAACGATACCGAGTGTACCTTCCCGCGCTCGCGCACCTGGAGC      600
KF786302.1      AACGTCAACCGCAAGATCAGCGATGCCGAGTGTACCTTCCCGCGCTCGCGCACCTGGAGC      600
KC812122.1      AACGTCAACCGCAAGATCAGCGATGCCGAGTGTACCTTCCCGCGCTCGCGCACCTGGAGC      600
                ******************* **** ******* ***************************

AY442273.1      CGGGGCGACCGGTTGTTCTGCGATTCGCCGAACATTTCGCTGGTCTACCGGGTTAACCTG      660
GU229025.1      CGGGGCGACCGGTTGTTCTGCGATTCGCCGAACATTTCGCTGGTCTACCGGGTTAACCTG      660
LR963141.1      CGGGGCGACCGGTTGTTCTGCGATTCGCCGAACATTTCGCTGGTCTACCGGGTTAACCTG      660
KC812121.1      CGGGGTGATCGGCTGTTCTGCGATTCGCCGAACATCTCGCTGGTCTACCGGGTCAACCTG      660
KC812120.1      CGGGGTGATCGGCTGTTCTGCGATTCGCCGAACATCTCGCTGGTCTACCGGGTCAACCTG      660
U81555.1        CGGGGCGACCGGCTGTTCTGCGATTCGCCGAACATCTCGCTGGTTTACCGGGTCAACCTG      660
KF786302.1      CGGGGCGACCGGCTGTTCTGCGATTCGCCGAACATCTCGCTGGTTTACCGGGTCAACCTG      660
KC812122.1      CGGGGCGACCGGCTGTTCTGCGATTCGCCGAACATCTCGCTGGTTTACCGGGTCAACCTG      660
                ***** ** *** ********************** ******** ******** ******

AY442273.1      GAGCGCTCCCTGCAATTTGGCAACACCGGCTCCGCCACGCCGGATGCCAAGATAGTGCGG      720
GU229025.1      GAGCGCTCCCTGCAATTTGGCAACACCGGCTCCGCCACGCCGGATGCCAAGATAGTGCGG      720
LR963141.1      GAGCGCTCCCTGCAATTTGGCAACACCGGCTCCGCCACGCCGGATGCCAAGATAGTGCGG      720
KC812121.1      GAGCGCTCCCTGCAATTTGGCAACACCGGCTCCGCCACGCCGGATGCCAAGATAGTGCGG      720
KC812120.1      GAGCGCTCCCTGCAATTTGGCAACACCGGCTCCGCCACGCCGGATGCCAAGATAGTGCGG      720
U81555.1        GAGCGCTCCCTGCAATTTGGCAACACCGGCTCCGCCACGCCGGATGCCAAGATAGTGCGG      720
KF786302.1      GAGCGCTCCCTGCAATTTGGCAACACCGGCTCCGCCACGCCGGATGCCAAGATAGTGCGG      720
KC812122.1      GAGCGCTCCCTGCAATTTGGCAACACCGGCTCCGCCACGCCGGATGCCAAGATAGTGCGG      720
                ************************************************************

AY442273.1      ATCTCGCTGGACGAAGAGTCGGCCGGTGCTGGCATCCAGCTCAACGAGGATCTGAGCTGG      780
GU229025.1      ATCTCGCTGGACGAAGAGTCGGCCGGTGCTGGCATCCAGCTCAACGAGGATCTGAGCTGG      780
LR963141.1      ATCTCGCTGGACGAAGAGTCGGCCGGTGCTGGCATCCAGCTCAACGAGGATCTGAGCTGG      780
KC812121.1      ATCTCGCTGGACGAAGAGTCGGCCGGTGCCGGCATCCAGCTCAACGAGGATCTGAGCTGG      780
KC812120.1      ATCTCGCTGGACGAAGAGTCGGCCGGTGCCGGCATCCAGCTCAACGAGGATCTGAGCTGG      780
U81555.1        ATCTCGCTGGATGAAGAGTCGGCCGGTGCCGGTATCCAGCTCAACGAGGAGCTGAGCTGG      780
KF786302.1      ATCTCGCTGGACGAAGAGTCGGCCGGTGCCGGTATCCAGCTCAACGAGGAGCTGAGCTGG      780
KC812122.1      ATCTCGCTGGACGAAGAGTCGGCCGGTGCCGGTATCCAGCTCAACGAGGAGCTGAGCTGG      780
                *********** ***************** ** ***************** *********
```

```
AY442273.1      AGCGAGAACATCGCGGACTACCTGCTGCTGGATGGCTGGGCCCGCGACTATGCCACCGAT      840
GU229025.1      AGCGAGAACATCGCGGACTACCTGCTGCTGGATGGCTGGGCCCGCGACTATGCCACCGAT      840
LR963141.1      AGCGAGAACATCGCGGACTACCTGCTGCTGGATGGCTGGGCCCGCGACTATGCCACCGAT      840
KC812121.1      AGCGAGAACATCGCGGACTACCTGCTGCTGGATGGCTGGGCCCGCGACTATGCCACCGAT      840
KC812120.1      AGCGAGAACATCGCGGACTACCTGCTGCTGGATGGCTGGGCCCGCGACTATGCCACCGAT      840
U81555.1        AGCGAGAACATTACGGACTATCTCCTGCTGGACGGCTGGGCCCGCGACTATGCCACCGAT      840
KF786302.1      AGCGAGAACATTGCGGACTATCTGCTGCTGGACGGCTGGGCCCGCGACTATGCCACCGAT      840
KC812122.1      AGCGAGAACATTGCGGACTATCTGCTGCTGGACGGCTGGGCCCGCGACTATGCCACCGAT      840
                ***********  ******* ** ******** ***************************

AY442273.1      GCCATCGCCCAGGATTACCGCTTCAGCATCGAGGCGTCCAACACCAAGGCGGCGGTGCTC      900
GU229025.1      GCCATCGCCCAGGATTACCGCTTCAGCATCGAGGCGTCCAACACCAAGGCGGCGGTGCTC      900
LR963141.1      GCCATCGCCCAGGATTACCGCTTCAGCATCGAGGCGTCCAACACCAAGGCGGCGGTGCTC      900
KC812121.1      GCCATCGCCCAGGATTATCGCTTCAGCATCGAGGCGTCCAACACCAAGGCGGCGGTGCTC      900
KC812120.1      GCCATCGCCCAGGATTATCGCTTCAGCATCGAGGCGTCCAACACCAAGGCGGCGGTGCTC      900
U81555.1        GCCATCGCCCAGGATTACCGCTTCAGCATCGAGGCGTCCAACACCAAGGCTGCGGTGCTC      900
KF786302.1      GCCATCGCCCAGGATTACCGCTTCAGCATCGAGGCGTCCAACACCAAGGCTGCGGTGCTC      900
KC812122.1      GCCATCGCCCAGGATTACCGCTTCAGCATCGAGGCGTCCAACACCAAGGCTGCGGTGCTC      900
                ***************** ********************************* ********

AY442273.1      AAGAGCCTGCCGACCAACCCCAACAGCAAGTACGAGCATCGCGAGAGCTCGGGCTTCGAG      960
GU229025.1      AAGAGCCTGCCGACCAACCTCAACAGCAAGTACGAGCATCGCGAGATCTCGGGCTTCGAG      960
LR963141.1      AAGAGCCTGCCGACCAACCTCAACAGCAAGTACGAGCATCGCGAGAGCTCGGGCTTCGAG      960
KC812121.1      AAGAGCCTGCCGACCAACCTCAACAGCAAGTATGAGCATCGCGAGATCTCGGGTTTCGAG      960
KC812120.1      AAGAGCCTGCCGACCAACCTCAACAGCAAGTACGAGCATCGCGAGATCTCGGGTTTCGAG      960
U81555.1        AAGAGCCTGCCGACCAACCTCAACAGCAAGTACGAGCATCGCGAGATCTCGGGTTTCGAG      960
KF786302.1      AAGAGCCTGCCGACCAACCTCAACAGCAAGTACGAGCATCGCGAGATCTCGGGTTTCGAG      960
KC812122.1      AAGAGCCTGCCGACCAACCTCAACAGCAAGTACGAGCATCGCGAGATCTCGGGTTTCGAG      960
                ******************* ************ ************* ****** ******

AY442273.1      GTGGGGGTCACCGGCGGGGTCGAGGTGAACAAGGATGGGCCCAAGGCCAAGCTGGAGGCG      1020
GU229025.1      GTGGGGGTCACCGGCGGGGTCGAGGTGAACAAGGATGGGCCCAAGGCCAAGCTGGAGGCG      1020
LR963141.1      GTGGGGGTCACCGGCGGGGTCGAGGTGAACAAGGATGGGCCCAAGGCCAAGCTGGAGGCG      1020
KC812121.1      GTGGGGGTCACCGGCGGGGTCGAGGTGAACAAGGATGGGCCCAAGGCCAAGCTGGAGGCG      1020
KC812120.1      GTGGGGGTCACCGGAGGGGTCGAGGTGAACAAGGATGGGCCCAAGGCCAAGCTGGAGGCG      1020
U81555.1        GTGGGGGTCACTGGCGGGGTCGAGGTGAACAAGGATGGGCCCAAGGCCAAGCTGGAGGCG      1020
KF786302.1      GTGGGGGTCACCGGCGGGGTGGAGGTGAACAAGGATGGGCCCAAGGCCAAGCTGGAGGCG      1020
KC812122.1      GTGGGGGTCACCGGCGGGGTGGAGGTGAACAAGGATGGGCCCAAGGCCAAGCTGGAGGCG      1020
                *********** ** ***** ***************************************

AY442273.1      TCGGCCAAGTTCAGCCAGCAGCGTCAGCTCGCCTACAACACCCAGGATTACCGGGTTGAA      1080
GU229025.1      TCGGCCAAGTTCAGCCAGCAGCGTCAGCTCGCCTACAACACCCAGGATTACCGGGTTGAA      1080
LR963141.1      TCGGCCAAGTTCAGCCAGCAGCGTCAGCTCGCCTACAACACCCAGGATTACCGGGTTGAA      1080
KC812121.1      TCGGCCAAGTTCAGCCAGCAGCGCCAGCTTGCCTACAACACCCAGGATTACCGGGTCGAA      1080
KC812120.1      TCGGCCAAGTTCAGCCAGCAGCGCCAGCTTGCCTACAACACCCAGGATTACCGGGTCGAA      1080
U81555.1        TCGGCCAAGTTCAGCCAGCAGCGCCAGCTCGCCTACAACACCCAGGATTACCGGGTCGAA      1080
KF786302.1      TCGGCCAAGTTCAGCCAGCAGCGCCAGCTCGCCTACAACACCCAGGATTACCGGGTTGAA      1080
KC812122.1      TCGGCCAAGTTCAGCCAGCAGCGCCAGCTCGCCTACAACACCCAGGATTACCGGGTTGAA      1080
                *********************** ***** ************************** ***
```

```
AY442273.1        CGCTCGGCGCCGAGCGCCCAGAAGGTGAGTTTCAGCTGGGTGCGGGATCAATATGCGACC        1140
GU229025.1        CGCTCGGCGCCGAGCGCCCAGAAGGTGAGTTTCAGCTGGGTGCGGGATCAATATGCGACC        1140
LR963141.1        CGCTCGGCGCCGAGCGCCCAGAAGGTGAGTTTCAGCTGGGTGCGGGATCAATATGCGACC        1140
KC812121.1        CGCTCGGCGCCGAGCGCCCAGAAGGTGAGTTTCAGCTGGGTGCGCGATCAGTACGCGACC        1140
KC812120.1        CGCTCGGCGCCGAGCGCCCAGAAGGTGAGTTTCAGCTGGGTGCGCGATCAATACGCGACC        1140
U81555.1          CGCTCGGCGCCGAGTGCCCAGAAGGTGAGTTTCAGCTGGGTGCGCGATCAATACGCGACC        1140
KF786302.1        CGCTCGGCGCCGAGCGCCCAGAAGGTGAGTTTCAGCTGGGTGCGCGATCAGTACGCGACC        1140
KC812122.1        CGCTCGGCGCCGAGCGCCCAGAAGGTGAGTTTCAGCTGGGTGCGCGATCAGTACGCGACC        1140
                  ************** ***************************** ***** ** ******

AY442273.1        GCGGAGTCCCTGCTCTCCTCCAAGACGGCCACCGTCTGGGGCATGGGCTACGACGTGGAT        1200
GU229025.1        GCGGAGTCCCTGCTCTCCTCCAAGACGGCCACCGTCTGGGGCATGGGCTACGACGCGGAT        1200
LR963141.1        GCGGAGTCCCTGCTCTCCTCCAAGACGGCCACCGTCTGGGGCATGGGCTACGACGTGGAT        1200
KC812121.1        GCGGAGTCCCTGCTCTCCTCCAAGACGGCCACCGTCTGGGGCATGGGGTACGATGTGGAT        1200
KC812120.1        GCGGAGTCCCTGCTCTCCTCCAAGACGGCCACCGTCTGGGGCATGGGCTACGACGTGGAT        1200
U81555.1          GCGGAGTCCCTGCTCTCCTCCAAGACTGCCACCGTCTGGGGCATGGGGTACGATGTAGAT        1200
KF786302.1        GCAGAGTCCCTGCTCTCCTCCAAGACGGCCACCGTCTGGGGCATGGGCTACGACGTGGAT        1200
KC812122.1        GCAGAGTCCCTGCTCTCCTCCAAGACGGCCACCGTCTGGGGCATGGGCTACGACGTGGAT        1200
                  ** *********************** ******************** ***** *  ***

AY442273.1        CACAACCGTATCCAGCCGCTCAGCTACAAGGGGTTCGTGCCGAATCTGGACGTCATCTAC        1260
GU229025.1        CACAACCGTATCCAGCCGCTCAGCTACAAGGGGTTCGTGCCGAATCTGGACGTCATCTAC        1260
LR963141.1        CACAACCGTATCCAGCCGCTCAGCTACAAGGGGTTCGTGCCGAATCTGGACGTCATCTAC        1260
KC812121.1        CACAACCGCATCCAGCCGCTCAGCTACAAGGGGTTCGTGCCGAATCTGGACGTCATCTAC        1260
KC812120.1        CACAACCGCATCCAGCCGCTCAGCTACAAGGGGTTCGTGCCGAATCTGGACGTCATCTAC        1260
U81555.1          CACAACCGCATCCAGCCGCTCAGCTACAAGGGGTTCGTGCCAAACCTGGATGTCATCTAC        1260
KF786302.1        CACAACCGCATCCAGCCGCTCAGCTACAAGGGGTTCGTGCCGAATCTGGATGTCATCTAC        1260
KC812122.1        CACAACCGCATCCAGCCGCTCAGCTACAAGGGGTTCGTGCCGAATCTGGATGTCATCTAC        1260
                  ******** ******************************** ** ***** *********

AY442273.1        AAGGCGGCACCGGACGAGACGGGCAGCACCGAGTTCAAGATCGACTC-TCGGTCAACATC        1319
GU229025.1        AAGGCGGCACCGGACGAGACGGGCAGCACCGAGTTCAAGATCGACTCCTCGGTCAACATC        1320
LR963141.1        AAGGCGGCACCGGACGAGACGGGCAGCACCGAGTTCAAGATCGACTCCTCGGTCAACATC        1320
KC812121.1        AAGGCGGCACCGGACGAGACGGGCAGCACCGAGTTCAAGATCGACTCCTCGGTCAACATC        1320
KC812120.1        AAGGCGGCACCGGACGAGACGGGCAGCACCGAGTTCAAGATCGACTCCTCGGTCAACATC        1320
U81555.1          AAGGCGGCGCCGGACGAGACGGGCAGCACCGAGTTCAAGATCGACTCCTCGGTCAACATC        1320
KF786302.1        AAGGCGGCGCCGGACGAGACGGGCAGCACCGAGTTCAAGATCGACTCCTCGGTCAACATC        1320
KC812122.1        AAGGCGGCGCCGGACGAGACGGGCAGCACCGAGTTCAAGATCGACTCCTCGGTCAACATC        1320
                  ******** ************************************** ************

AY442273.1        CGCCCCATCTACACCGGCATCTACAAGCACTACTACGTGGTGGGGGCCCATGTCTCCTTC        1379
GU229025.1        CGCCCCATCTACACCGGCATCTACAAGCACTACTACGTGGTGGGGGCCCATGTCTCCTTC        1380
LR963141.1        CGCCCCATCTACACCGGCATCTACAAGCACTACTACGTGGTGGGGGCCCATGTCTCCTTC        1380
KC812121.1        CGCCCCATCTACACCGGCATCTACAAGCACTACTACGTGGTGGGGGCCCATGTCTCCTTC        1380
KC812120.1        CGCCCCATCTACACCGGCATCTACAAGCACTACTACGTGGTGGGGGCCCATGTCTCCTTC        1380
U81555.1          CGCCCCATTTATACCGGCATCTACAAGCACTACTACGTGGTGGGGGCCCATGTCTCCTTC        1380
KF786302.1        CGCCCCATCTACACCGGCATCTACAAGCACTACTACGTGGTGGGGGCCCATGTCTCCTTC        1380
KC812122.1        CGCCCCATCTACACCGGCATCTACAAGCACTACTACGTGGTGGGGGCCCATGTCTCCTTC        1380
                  ******** ** ************************************************
```

```
AY442273.1      CAGGGCTTTGAAGATACCGACAAGCGTAGACGGGTGA-----------------------    1416
GU229025.1      CAGGGCTTTGAAGATACCGACAAGCGTAGACGGGTGACGGCGTCCACCAGCTTCAAAGTG    1440
LR963141.1      CAGGGCTTTGAAGATACCGACAAGCGTAGACGGGTGACGGCGTCCACCAGCTTCAAAGTG    1440
KC812121.1      CAGGGCTTCGAAGATACCGACAAGCGCAGACGGGTGACGGCATCCACCAGCTTCAAGGTG    1440
KC812120.1      CAGGGCTTTGAAGATACCGACAAGCGCAGACGGGTGACGGCGTCCACCAGCTTCAAGGTG    1440
U81555.1        CAGGGTTTCGAGGATACCGACAAGCGCAGACGGGTGACGGCGTCCACCAGCTTCAAGGTG    1440
KF786302.1      CAGGGCTTCGAAGATACCGACAAGCGCAGACGGGTGACGGCGTCCACCAGCTTCAAGGTG    1440
KC812122.1      CAGGGCTTCGAAGATACCGACAAGCGCAGACGGGTGACGGCGTCCACCAGCTTCAAGGTG    1440
                ***** ** ** ************** **********

AY442273.1      ------------------------------------------------------------    1416
GU229025.1      GACTGGAACCACCCGGTGTTTACCGGCGGTCGCCCGGTCAACCTGCAACTGGGGGGTTTT    1500
LR963141.1      GACTGGAACCACCCGGTGTTTACCGGCGGTCGCCCGGTCAACCTGCAACTGGGGGGTTTT    1500
KC812121.1      GACTGGAACCACCCGGTGTTTACCGGCGGTCGCCCGGTCAACCTGCAGCTGGGGGGCTTT    1500
KC812120.1      GACTGGAACCACCCGGTGTTTACCGGCGGTCGCCCGGTCAACCTGCAGCTGGGGGGCTTT    1500
U81555.1        GACTGGAACCACCCGGTGTTTACCGGCGGTCGCCCGGTCAACCTGCAACTGGGGGGCTTT    1500
KF786302.1      GACTGGAATCACCCGGTGTTTACCGGCGGTCGCCCGGTCAACCTGCAGCTGGGGGGCTTT    1500
KC812122.1      GACTGGAATCACCCGGTGTTTACCGGCGGTCGCCCGGTCAACCTGCAGCTGGGGGGCTTT    1500

AY442273.1      ------------------------------------------------------------    1416
GU229025.1      GACAACCGCTGTCTGAGCGCGGATGCCAACCATGGGCTGAGCGCGGTGACCTGTGACGAG    1560
LR963141.1      GACAACCGCTGTCTGAGCGCGGATGCCAACCATGGGCTGAGCGCGGTGACCTGTGACGAG    1560
KC812121.1      GACAACCGCTGCCTGAGCGCGGATGCCAACCATGGTCTGAGCGCGGTAACCTGTGACGAG    1560
KC812120.1      GACAACCGCTGCCTGAGCGCGGATGCCAACCATGGTCTGAGCGCGGTGACCTGTGACGAG    1560
U81555.1        GACAATCGCTGTCTGAGCGCGGATGCCAACCATGGTCTGAGCGCGGTAACCTGTGACGAG    1560
KF786302.1      GACAACCGCTGCCTGAGCGCGGATGCCAACCATGGTCTGAGTGCGGTGACCTGTGACGAG    1560
KC812122.1      GACAACCGCTGCCTGAGCGCGGATGCCAACCATGGTCTGAGTGCGGTGACCTGTGACGAG    1560

AY442273.1      ------------------------------------------------------------    1416
GU229025.1      ACCTCGGCCGCCCAGTCCTTCATCTATGACCAGTACGGCCGCTATGTCAGCGCGCAGGAT    1620
LR963141.1      ACCTCGGCCGCCCAGTCCTTCATCTATGACCAGTACGGCCGCTATGTCAGCGCGCAGGAT    1620
KC812121.1      ACCTCGGCCGCCCAGTCTTTCATCTATGACCAGTACGGCCGCTATGTCAGCGCGCAGGAT    1620
KC812120.1      ACCTCGGCCGCCCAGTCCTTCATCTATGACCAGTACGGCCGCTATGTCAGCGCGCAGGAT    1620
U81555.1        ACCTCGGCCGCCCAGTCCTTCATCTATGACCAGTACGGCCGCTATGTCAGCGCGCAGGAT    1620
KF786302.1      ACCTCGGCTGCCCAGTCCTTCATCTATGACCAGTACGGCCGCTATGTCAGCGCGCAGGAT    1620
KC812122.1      ACCTCGGCTGCCCAGTCCTTCATCTATGACCAGTACGGCCGCTATGTCAGCGCGCAGGAT    1620

AY442273.1      ------------------------------------------------------------    1416
GU229025.1      ACCCGCCGCTGTCTGGACGGCAACAACCTCGGCCAGCTGCAGAGCTGCAGTCTGAGCCTG    1680
LR963141.1      ACCCGCCGCTGTCTGGACGGCAACAACCTCGGCCAGCTGCAGAGCTGCAGTCTGAGCCTG    1680
KC812121.1      ACCCGCCGCTGCCTGGACGGCAACAACCTCGGCCAGCTG---------------------    1659
KC812120.1      ACCCGCCGCTGCCTGGACGGCAACAACCTCGGCCAGCTG---------------------    1659
U81555.1        ACCCGCCGCTGCCTGGATGGCAACAACCTCGGCCAGCTGCAGAGTTGCAGCCTGAGCCTG    1680
KF786302.1      ACCCGCCGCTGCCTGGATGGCAACAACCTTGGCCAGCTGCAGAGCTGCAGCCTGAGCCTG    1680
KC812122.1      ACCCGCCGCTGCCTGGATGGCAACAACCTTGGCCAGCTG---------------------    1659
```

```
AY442273.1     ------------------------------------------------------------     1416
GU229025.1     GGTCAGCGCTGGGAGTGGAAAGCGGACAGCGATGCACTCAGCAACCTGAGTGCCCACCAG     1740
LR963141.1     GGTCAGCGCTGGGAGTGGAAAGCGGACAGCGATGCACTCAGCAACCTGAGTGCCCACCAG     1740
KC812121.1     ------------------------------------------------------------     1659
KC812120.1     ------------------------------------------------------------     1659
U81555.1       GGTCAGCGCTGGGAGTGGAAAGCGGACAGCGATGCGCTCAGCAATCTGAGTGCCCACCAG     1740
KF786302.1     GGTCAGCGCTGGGAGTGGAAAGCGGACAGCGATGCGCTCAGCAACCTGAGTGCCCACCAG     1740
KC812122.1     ------------------------------------------------------------     1659


AY442273.1     ------------------------------------------------------------     1416
GU229025.1     CTGCTGGGTCATGACAAGCAGAGCGGGGCGCTGGGGCTCTACGACGAGAACGGCAACCCG     1800
LR963141.1     CTGCTGGGTCATGACAAGCAGAGCGGGGCGCTGGGGCTCTACGACGAGAACGGCAACCCG     1800
KC812121.1     ------------------------------------------------------------     1659
KC812120.1     ------------------------------------------------------------     1659
U81555.1       CTGCTCGGTCATGACAAGCAGAGCGGGGCGCTAGGGCTCTACGACGAGAACGGCAACCTG     1800
KF786302.1     CTGCTGGGTCATGACAAGCAGAGCGGAGCGCTGGGGCTCTACGACGAGAACGGCAATCCG     1800
KC812122.1     ------------------------------------------------------------     1659

AY442273.1     ------------------------------------------------------------     1416
GU229025.1     CAGAATGTGAGCGTACGGACCCTGACCTCCTACACCCGCATCTTCGGGCCACCGGCCAGT     1860
LR963141.1     CAGAATGTGAGCGTACGGACCCTGACCTCCTACACCCGCATCTTCGGGCCACCGGCCAGT     1860
KC812121.1     ------------------------------------------------------------     1659
KC812120.1     ------------------------------------------------------------     1659
U81555.1       CAGAATGTGAGCCTACGGACCCTGACCTCCTACACCCGTATCTTCGGGCCACCGGCCAGT     1860
KF786302.1     CAGAATGTGAGCGTACGGACCCTGACCTCCTACACCCGCATCTTCGGGCCACCGGCCAGC     1860
KC812122.1     ------------------------------------------------------------     1659


AY442273.1     ------   1416
GU229025.1     CACTGA   1866
LR963141.1     CACTGA   1866
KC812121.1     ------   1659
KC812120.1     ------   1659
U81555.1       CACTGA   1866
KF786302.1     CACTGA   1866
KC812122.1     ------   1659
```

Figure S5. The aligned results and the conserved regions of *A. hydrophila hlyA* gene. The sequences highlighted with green and yellow were primer HF3 and HR3, respectively. The sequences with the red underline and blue underline were used to design crRNA HCR1 and HCR2, respectively. *: indicates that the nucleotide is conserved among these sequences.

```
AF443394.1    TCCATCAAGGTAAGCAACTTCTCATATACCCTGGAACCCGACACCTTCAGCCATGGTCAG    60
AB109093.1    TCCATCAAGGTAAGCAACTTCTCATATACCCTGGAACCCGACTCCTTCAGCCATGGTCAG    60
EF034117.1    TCCATCAAGGTAAGCAACTTCTCATATACCCTGGAACCCGATTCCTTCAGCCATGGTCAG    60
CP080043.1    TCCATCAAGGTGAGCAATTTTGCCTACAACCTGGATCCTGACAGTTTCAAGCATGGCGAT    60
CP051883.1    TCCATCAAGGTGAGCAATTTTGCCTACAACCTGGATCCTGACAGTTTCAAGCATGGCGAT    60
HQ425626.1    GCCATCAAGGTCAGCAATTTTGCGTACAACCTGGACCCTGACAGCTTCAAACATGGTGAT    60
KF955964.1    GCCATCAAGGTCAGCAATTTTGCGTACAACCTGGACCCTGACAGTTTCAAACATGGTGAC    60
               ********** ***** **  * ** * ****** ** **    ****  *****  *

AF443394.1    GTCACCGAGAGCGGCAAGCAGCTGGTGAAGACCATCACGGCCAATGCGACCAACTACACC    120
AB109093.1    GTCACCGAGAGCGGCAAGCAGCTGGTCAAAACCATCACGGCCAATGCGACCAACTACACC    120
EF034117.1    GTCACCGAGAGCGGCAAGCAGCTGGTCAAGACCATCTCGGCCAATGCGACCAACTACACG    120
CP080043.1    GTGACCCAGTCCGACCGCCAACTGGTCAAGACGGTGGTGGGCTGGGCTATCAACGACAGC    120
CP051883.1    GTGACCCAGTCCGACCGCCAACTGGTCAAGACGGTGGTGGGGTGGGCTATCAACGACAGC    120
HQ425626.1    GTGACCCAGTCTGATCGCCAGCTGGTCAAGACGGTGGTGGGCTGGGCGATCAACGACAGC    120
KF955964.1    GTGACCCAGTCTGATCGCCAACTGGTCAAGACGGTGGTGGGCTGGGCGATCAACGACAGC    120
              ** *** **   *     ** ***** ** **  *   **     ** * **** ***

AF443394.1    GACCAGCCC---CAGCAGGTGGTGGTGACCCTGATGTACGACAAGGCCACCAACTGGTCG    177
AB109093.1    GACCTGCCC---CAGCAGGTGGTGGTGACCCTGAAGTACGACAAGGCCACCAACTGGTCG    177
EF034117.1    GATCTGCCT---CAGCAGGTGGTGGTGACCCTGAAGTACGACAAGGCCACCAACTGGTCG    177
CP080043.1    GACACCCCTCAATCCGGTTATGACGTCACCCTGCGCTACGACACCGCCACCAACTGGTCC    180
CP051883.1    GACACCCCTCAATCCGGTTATGACGTCACCCTGCGCTACGACACCGCCACCAACTGGTCC    180
HQ425626.1    GACACCCCGCAATCCGGCTATGATGTCACCCTGCGTTACGATACCGCCACCAACTGGTCC    180
KF955964.1    GACACCCCGCAATCCGGTTATGATGTCACCCTGCGTTACGATACCGCCACCAACTGGTCC    180
              **    **             *  ** ******   ***** *  **************

AF443394.1    AAGACCGACACCTACTGTCTGAGCGAGAAGGTGACCACCAAGAACAAGTTTCAGTG    233
AB109093.1    AAAACCGATACCTACAGCCTGAGCGAGAAGGTGACCACCAAGAACAAGTTTCAGTG    233
EF034117.1    AAAACCGATACCTACAGCCTGAGCGAGAAGGTGACCACCAAGAACAAGTTTCAGTG    233
CP080043.1    AAGACCAACACCTATGGCCTGAGCGAGAAGGTGACCACCAAGAACAAGTTCAAGTG    236
CP051883.1    AAGACCAACACCTATGGCCTGAGCGAGAAGGTGACCACCAAGAACAAGTTCAAGTG    236
HQ425626.1    AAGACCAATACCTATGGCCTGAGCGAGAAGGTGACCACCAAGAACAAGTTCAAGTG    236
KF955964.1    AAGACCAATACCTATGGCCTGAGCGAGAAGGTGACCACCAAGAACAAGTTCAAGTG    236
              ** *** * *****  * ********************************  ****
```

Figure S6. The alignment of *A. hydrophila aerA* genes with the *aerA* sequences from other species of *Aeromonas*. The sequences highlighted with green and yellow were primer AF5 and AR5, respectively. The sequences with the red underline and blue underline were used to design crRNA ACR1 and ACR2, respectively. CAAA: the PAM of ACR1; TTTC: the PAM of ACR2. HQ425626.1: *A. hydrophila aerA* gene; KF955964.1: *A. hydrophila aerA* gene; CP080043.1: *A. salmonicida aerA* gene; CP051883.1: *A. salmonicida aerA* gene; AB109093.1: *A. veronii aerA* gene; EF034117.1: *A. veronii aerA* gene; AF443394.1: *A. sobria aerA* gene. *: indicates that the nucleotide is conserved among these sequences.

```
LC200779.1      CACGTGGCCTTCTACCTCAACGTCAACCGCAAGATCAGCGATGCCGAGTGCACCTTCCCG    60
GU229025.1      CACGTGGCCTTCTACCTCAACGTCAACCGCAAGATCAGCGATGCCGAGTGCACCTTCCCG    60
KU845732.1      CACGTGGCCTTCTACCTCAACGTCAACCGCAAGATCAGCGATGCCGAGTGCACCTTCCCG    60
CP000644.1      CACGTGGCTTTTTATCTCAACGTCAATCGCAAGATCAGCGATGGCGAGTGTACCTTCCTG    60
X65049.1        --CGGGCTTTTTATCTCAAGCTCAATCGCAAGATCAGCGTGGCCGAGTGTACCTTCCCG    58
                  ** *** ** ** *****  **** *************  * ****** ******* *

LC200779.1      CGCTCGCGCACCTGGAATCGGGGTGATCGGCTGTTCTGCGATTCGCCGAACATCTCGCTG    120
GU229025.1      CGCTCGCGCACCTGGAGCCGGGGCGACCGGTTGTTCTGCGATTCGCCGAACATTTCGCTG    120
KU845732.1      CGCTCGCGCACCTGGAGCCGGGGCGATCGGCTGTTCTGCGATTCGCCGAACATCTCGCTG    120
CP000644.1      CGCTCACGCACCTGGGATCGGGGGGCTCGCCTCTTTTGTGACTCGCCAAACATCTCGCTG    120
X65049.1        CGCTCACGCACCTGGGATCGGGGGGATCGCCTCTTTTGTGACTCGCCAAACATCTCGCTG    118
                ***** *********   ***** *  **  * ** ** ** ***** ***** ******

LC200779.1      GTCTACCGGGTCAACCTGGAGCGCTCCCTGCAATTTGGCAACACCGGTTCCGCCACGCCG    180
GU229025.1      GTCTACCGGGTTAACCTGGAGCGCTCCCTGCAATTTGGCAACACCGGCTCCGCCACGCCG    180
KU845732.1      GTCTACCGGGTCAACCTGGAGCGCTCCCTGCAATTTGGCAACACCGGTTCCGCCACGCCG    180
CP000644.1      GTCTATCGGGTGAATCTGGAGCGCTCCCTGCAATTTGGCAACACGGGGTCAGCTACGCCG    180
X65049.1        GTGTATCGGGTGAATCTGGAGCGCTCCCTGCAATTTGGCAACACGGGGTCAGCTACGCCG    178
                ** ** ***** ** ***************************** ** ** ** ******

LC200779.1      GATGCCAAGATAGTGCGGATCTCGCTGGACGAAGAGTCGGCCGGTGCCGGCATCCAGCTC    240
GU229025.1      GATGCCAAGATAGTGCGGATCTCGCTGGACGAAGAGTCGGCCGGTGCTGGCATCCAGCTC    240
KU845732.1      GATGCCAAAATAGTGCGGATCTCGCTGGACGAAGAGTCGGCCGGTGCCGGCATCCAGCTC    240
CP000644.1      GATGCCAAGATAGTGCGGATCTCGCTGGATGACGAGTCGGCAGGTGCCGGTATTCAGCTC    240
X65049.1        GATGCCAAGATAGTGCGGATCTCGCTGGATGACGAGTCGGCAGGTGGCGGTATTCAGCTC    238
                ******** ******************** ** ******** ****  ** ** ******

LC200779.1      GATGCCAAGATAGTGCGGATCTCGCTGGACGAAGAGTCGGCCGGTGCCGGCATCCAGCTC    300
GU229025.1      AACGAGGATCTGAGCTGGAGCGAGAACATCGCGGACTACCTGCTGCTGGATGGCTGGGCC    300
KU845732.1      AACGAGGATCTCAGCTGGAGCGAGAACATCGCGGACTACCTGCTGCTGGACGGCTGGGCC    300
CP000644.1      AATGAGGACCTCACCTGGAGCGAGAACATCGCAGATTATCTGCTGCTCGATGGCTGGGCG    300
X65049.1        AATGAGGACCTCACCTGGAGCGAGAACATCGCAGATTATCTGCTGCTCGATGGCTGGGCG    298
                 * *   *  *     *** *  *     **  ** *           *    *  *

LC200779.1      CGCGACTATGCCACCGATGCCATCGCTCAGGATTATCGCTTCAGCATCGAGGCGTCCAAC    360
GU229025.1      CGCGACTATGCCACCGATGCCATCGCCCAGGATTACCGCTTCAGCATCGAGGCGTCCAAC    360
KU845732.1      CGTGACTATGCCACCGATGCCATCGCCCAGGATTATCGCTTCAGCATCGAGGCGTCCAAC    360
CP000644.1      CGGGACTATGCCACCGATGCCATCGCCCAGGATTATCGCTTCACCATCGACGCTTCCAAT    360
X65049.1        CGGGACTATGCCACCGATGCCATCGCCCAGGATTATCGCTTCACCATCGACGCTTCCAAT    358
                ** *********************** ******** ******* ****** ** *****

LC200779.1      ACCAAGG    367
GU229025.1      ACCAAGG    367
KU845732.1      ACCAAGG    367
CP000644.1      ACCAAGG    367
X65049.1        ACCAAGG    365
                *******
```

Figure S7. The alignment of *A. hydrophila hlyA* genes with the *hlyA* sequences from other species of *Aeromonas*. The sequences highlighted with green and yellow were primer HF3 and HR3, respectively. The sequences with the red underline and blue

underline were used to design crRNA HCR1 and HCR2, respectively. TTTC: the PAM of HCR1; TTTG: the PAM of HCR2. GU229025.1: *A. hydrophila hlyA* gene; CP000644.1: *A. salmonicida hlyA* gene; X65049.1: *A. salmonicida hlyA* gene; KU845732.1: *A. veronii hlyA* gene; LC200779.1: A. *sobria hlyA* gene. *: indicates that the nucleotide is conserved among these sequences.
